# StPedf: Cell trajectory inference of spatial transcriptomics via spatial proximity embedding and spatial density-adaptive fusion

**DOI:** 10.1371/journal.pcbi.1014346

**Published:** 2026-06-05

**Authors:** Yuan Zhang, Ziyan Sun, Zhixin Shi, Mengdi Nan, Yuhan Fu, Qing Ren, Jie Gao

**Affiliations:** School of Science, Jiangnan University, Wuxi, Jiangsu, China; Penn State University: The Pennsylvania State University, UNITED STATES OF AMERICA

## Abstract

Spatial transcriptomics is transforming our multidimensional understanding of cellular spatial organization and its functional mechanisms in processes such as development and disease by systematically resolving the spatial heterogeneity of gene expression within tissues. To delve deeper into the dynamic processes underlying spatial expression patterns, spatial trajectory inference integrates genetic and spatial information to reconstruct the spatial developmental trajectories of cells within tissues. This approach reveals the patterns of differentiation and dynamic changes as cellular states evolve continuously along spatial axes. However, existing methods often struggle to uniformly model the complex, nonlinear interactions between high-dimensional gene expression and spatial coordinates. Here, we introduce StPedf, whose core lies in employing a neural network with a masking mechanism to capture complex nonlinear interactions between high-dimensional genes and spatial positions. It further leverages spatial proximity information as a guiding cue, dynamically and adaptively adjusting the embedding of gene and spatial information and the weighting of spatial proximity information based on spatial density. This enables trajectory inference guided by spatial information. This enables optimal transport to derive intercellular transition matrices, reconstruct cellular differentiation trajectories, and construct pseudo-spatiotemporal maps. StPedf demonstrates superior performance over existing methods on five structurally distinct simulated datasets. Using StPedf, we successfully mapped distinct lineages in the spatial trajectories of telencephalon regeneration in the *Ambystoma mexicanum*, multiple malignant lineages expanding within primary tumors, and developmental spatial trajectories and pseudo-spatiotemporal maps in human dorsolateral prefrontal cortex (DLPFC). StPedf significantly enhances the accuracy and interpretability of spatial trajectory inference, providing critical technical support for revealing the dynamic patterns of cellular fate transitions within tissue microenvironments.

## Author summary

The formation of tissue structure depends on the coordinated changes in cell states and their spatial arrangement during development, regeneration, and disease. Understanding these processes requires not only identifying gene activity but also determining the location of cells and how their spatial organization changes. Spatial transcriptomics allows us to study gene expression in its native tissue context, but reconstructing cellular trajectories remains challenging due to the complex, nonlinear coupling between gene expression patterns and spatial positions. To address this, we present StPedf, a spatial trajectory inference method that integrates gene expression, spatial location, and neighborhood information. Built on a neural network framework, StPedf captures the nonlinear relationships between molecular features and tissue structure, and adaptively balances gene and spatial information through cell density, improving trajectory reconstruction. Across simulated datasets and multiple real biological datasets, StPedf outperforms existing methods in recovering spatial trajectories. The method reveals regeneration-associated trajectories in axolotl telencephalon, diverse expansion lineages in intrahepatic cholangiocarcinoma (ICC) dataset, and developmental trajectories in DLPFC. StPedf provides an accurate and interpretable tool for studying dynamic cell-state transitions in complex tissue microenvironments.

## 1. Introduction

Trajectory inference aims to reconstruct continuous biological processes from discrete single-cell data, serving as a core technology for deciphering cellular differentiation dynamics. Traditional trajectory inference methods (e.g., Monocle [1], Slingshot [2], PAGA [3], TSCAN [4], destiny [5], and scVelo [6]) have been widely applied in single-cell RNA sequencing (scRNA-seq) but suffer from fundamental limitations. First, these methods lack a spatial dimension and cannot reflect actual cell migration paths or the role of the spatial microenvironment. Cellular interactions in complex tissues often exhibit multicellular cooperative characteristics [7], making it difficult for trajectory inference based solely on non-spatial single-cell data to fully reconstruct the true evolutionary process. Second, their topological structures are simplistic, most methods effectively handle only linear or simple branching structures, struggling to accurately depict processes with complex spatial dynamics like brain regeneration, tumor metastasis, or embryonic development. Third, trajectory directionality is ambiguous. Although dynamic models like RNA velocity attempt to infer direction, they often generate non-physical reverse trajectories due to splicing noise. Breakthroughs in spatial transcriptomics have introduced new dimensions to trajectory inference. Spatial transcriptomics (ST) technologies (e.g., Stereo-seq [8], Slide-seq [9–10]) simultaneously capture gene expression and cellular spatial coordinates, theoretically revealing spatial cellular fate transitions. By integrating multimodal approaches such as eMCI [11] to further characterize cellular composition and potential cell–cell interactions within spatial domains, richer contextual information can be provided for subsequent spatial trajectory reconstruction. This offers unprecedented opportunities to decipher spatial heterogeneity in cellular states within tissues and map dynamic migration trajectories during regeneration, disease, and development [10].

At present, various cell trajectory inference approaches based on spatial transcriptomics data have been developed. These approaches can be broadly classified into two main categories. The first category involves distance-weighted fusion strategies, which integrate gene expression profiles with spatial distance information to infer trajectories. For example, stLearn [12] employs a pseudo-spatiotemporal algorithm (PSTS) that combines pseudo-temporal distances derived from gene expression and physical distances based on spatial coordinates, constructing a fused distance matrix via weighted averaging for use in diffusion-based pseudo-time trajectory inference. Spatrack [13] also adopts a distance-weighted fusion strategy, effectively combining spatial proximity and gene similarity to infer spatial cellular developmental pathways. The second category comprises spatially-aware dimensionality reduction embedding strategies. These approaches reduce the dimensionality of cells or specific multicellular spatial regions to generate low-dimensional embeddings that preserve both spatial and expression features, providing a foundation for subsequent trajectory inference. Cell-level embedding approaches are widely used, such as SpaceFlow [14], which utilizes a dual graph convolutional network and deep information maximization to learn embeddings retaining transcriptional similarity and spatial neighborhood structure, then applies diffusion pseudo-time algorithms to construct spatial trajectories. SEDR [15] employs a graph autoencoder with spatial attention mechanisms to learn low-dimensional representations enhancing resolution of complex spatial patterns, also using diffusion pseudo-time algorithms on embeddings for pseudo-spatiotemporal graph construction. SpatialPCA [16] builds upon probabilistic principal component analysis by modeling spatial correlations through a kernel matrix to incorporate spatial constraints, generating embeddings that preserve neighborhood relationships and interface directly with Slingshot for spatial trajectory inference. Methods for dimensionality reduction and embedding of multicellular spatial regions (niches) are less common, with ONTraC [17] being a representative example. This method treats multicellular niches as analytical units, constructing a niche network integrating spatial and cell-type information, applying graph convolutional encoding to extract low-dimensional spatial features, using graph pooling to identify niche clusters and build cluster networks, generating niche trajectories (NT), and finally mapping cell–niche associations to yield cell-level NT scores characterizing continuous tissue microenvironment variations.

Although the aforementioned methods represent significant progress in integrating spatial information, they exhibit clear limitations when inferring dynamic trajectories across multiple time points. As noted by Heitz et al.[18], existing algorithms either rely on rigid coupling between gene expression and space while ignoring tissue deformation or process each time point independently while neglecting intertemporal cellular continuity. Specifically, distance-weighted strategies are sensitive to tissue deformation, and their fixed-weight design cannot flexibly adapt to the varying importance of gene expression versus spatial constraints across different biological contexts. While dimensionality reduction and embedding methods can learn more robust feature representations, most fail to explicitly model the dynamic interplay between the temporal evolution of cellular states and spatial structure. Recent studies have explored optimal transport theory for cross-temporal slice alignment and trajectory reconstruction. For instance, PASTE [19,20] uses a hybrid Gromov-Wasserstein distance for slice registration, stVCR [21] introduces dynamic and unbalanced optimal transport algorithms to model cell displacement, and Spateo [22] utilizes morphometric vector fields to characterize continuous spatial structural changes. Optimal transport enables cost-minimizing mappings, intuitively quantifying differences between two distributions. It flexibly integrates diverse data as matching constraints—such as gene expression and spatial location information—to yield precise and interpretable correspondences. This supports high-precision analyses like trajectory inference and cellular communication. However, these approaches still face significant technical challenges in the joint optimization of spatial alignment and trajectory continuity, including high computational complexity, sensitivity to data quality, and limited generalization across datasets. Therefore, developing trajectory inference algorithms that can adapt to complex multi-temporal dynamic scenarios while preserving both spatial structure and cellular state continuity remains a critical research direction in spatial transcriptomics.

To overcome the aforementioned bottlenecks, we propose StPedf, a framework that integrates spatial proximity embedding with a spatial density-adaptive fusion mechanism. First, StPedf generates latent variable representations that fuse gene expression with spatial coordinates through a graph neural network architecture, effectively addressing the issue of insufficient integration between gene and spatial information in spatial transcriptomics. Second, StPedf reuses spatial proximity information as a guide, dynamically and adaptively adjusting the weight distribution between embeddings and spatial proximity based on spatial density. This overcomes the limitation of traditional methods in complex tissue structures, where balancing contributions from multiple information sources is inflexible, enabling adaptive weighting of multi-source information. Furthermore, StPedf explicitly constructs a cell transport map across time points based on optimal transport theory, fundamentally resolving the issue of disrupted cellular connections between consecutive time points. Finally, leveraging a unified latent variable representation, StPedf generates a cell migration velocity field, enabling quantitative and synchronized characterization of cellular differentiation directions. This approach addresses the challenges of consistency and interpretability in dynamic process inference. Results demonstrate that across five simulated datasets with distinct structures, StPedf exhibits significant competitive advantage over six mainstream trajectory inference algorithms. Experiments spanning scenarios such as brain regeneration in Western newts, tumor microenvironment evolution, and human DLPFC development (S1 Table) confirm StPedf’s efficacy in supporting in-depth analyses in developmental biology and disease research.

## 2. Results

### 2.1. Overview

**Fig 1:**
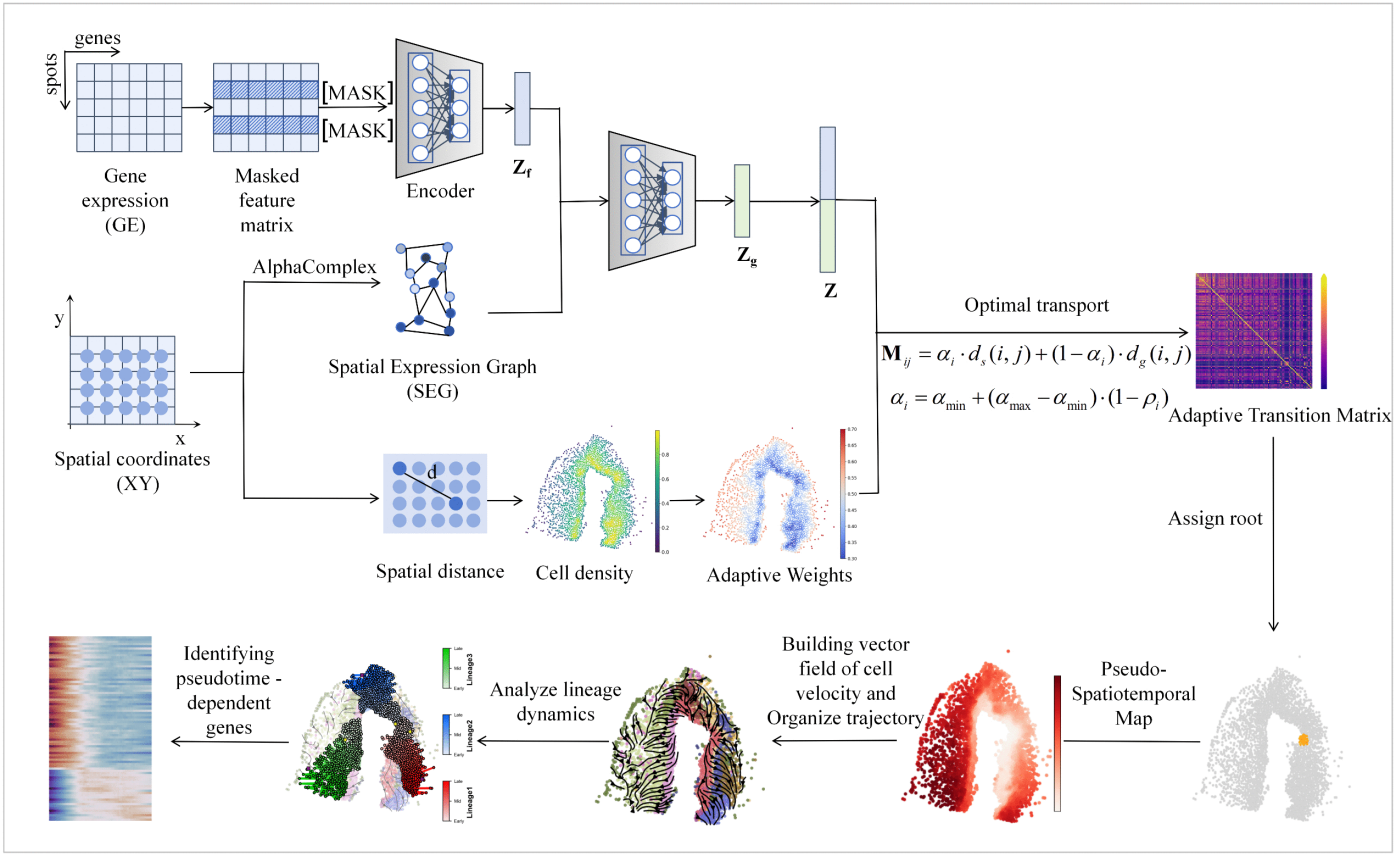
StPedf workflow diagram. StPedf takes the gene expression (GE) matrix and spatial coordinates (**XY**) as input. A masking strategy is first applied to the gene expression matrix, and the masked features are encoded to obtain the feature embedding $Z_{f}$. In parallel, spatial coordinates are used to construct the spatial expression graph (**SEG**) via Alpha Complex, from which the graph embedding $Z_{g}$is learned. The two embeddings are then integrated into a joint latent representation 𝐙. Based on this representation, StPedf computes a fused transport cost matrix in which $d_{f}\left(i,j\right)$ denotes the distance between spots/cells i and 𝐣 in the joint embedding space, and $d_{s}\left(i,j\right)$ denotes their Euclidean distance in the original spatial coordinate space. The relative contribution of the explicit spatial distance term is adaptively adjusted according to the local spatial density $\rho_{i}$, yielding the density-aware weight $\alpha_{i}$. Here, $\alpha_{i}$approaches $\alpha_{min}$in high-density regions and $\alpha_{max}$ in low-density regions. The resulting fused cost $M_{ij}$ is used to construct the adaptive transition matrix based on optimal transport. This transition matrix is then used to assign the root, generate the pseudo-spatiotemporal map, infer lineage dynamics, construct the cell-state velocity field, organize trajectories, and identify pseudotime-dependent genes.

The workflow of StPedf is illustrated in Fig 1. First, spatial transcriptomic gene expression data are input into a masked self-supervised deep autoencoder [23–24] to generate a latent representation 𝐙. Spatial coordinates from the same assay are used to construct a spatial expression map based on the α-complex. In this graph, nodes represent cells with their expression profiles, and edges denote spatial adjacency relationships between cells. A variational graph convolutional autoencoder (VGAE) [25] is then employed to fuse gene expression and spatial information into a joint embedding 𝐙. A density-adaptive strategy dynamically balances the weights between high-density regions, which are dominated by embedding features, and low-density regions, which are guided by spatial information, resulting in a merged representation $M_{𝐢𝐣}$. When the initial embedding shows trajectory tendencies, weighted spatial information enhances these patterns. When the embedding structure is disorganized, spatial information helps correct the distortion. By combining deep feature extraction with adaptive fusion, gene expression features and spatial constraints are integrated synergistically. Using optimal transport [26], a transition matrix is derived. We sort the cells based on the probability of their transfer from a reference cell, and then use the resulting sorting results to construct a cell velocity vector field. The path of least action [27] is used to identify the optimal path between initial and terminal cells, enabling end-to-end analysis from raw data to trajectory inference, lineage segmentation, and pseudotemporal gene identification. Technical details are provided in the Methods section.

### 2.2. Performance on different structural simulation datasets

**Fig 2:**
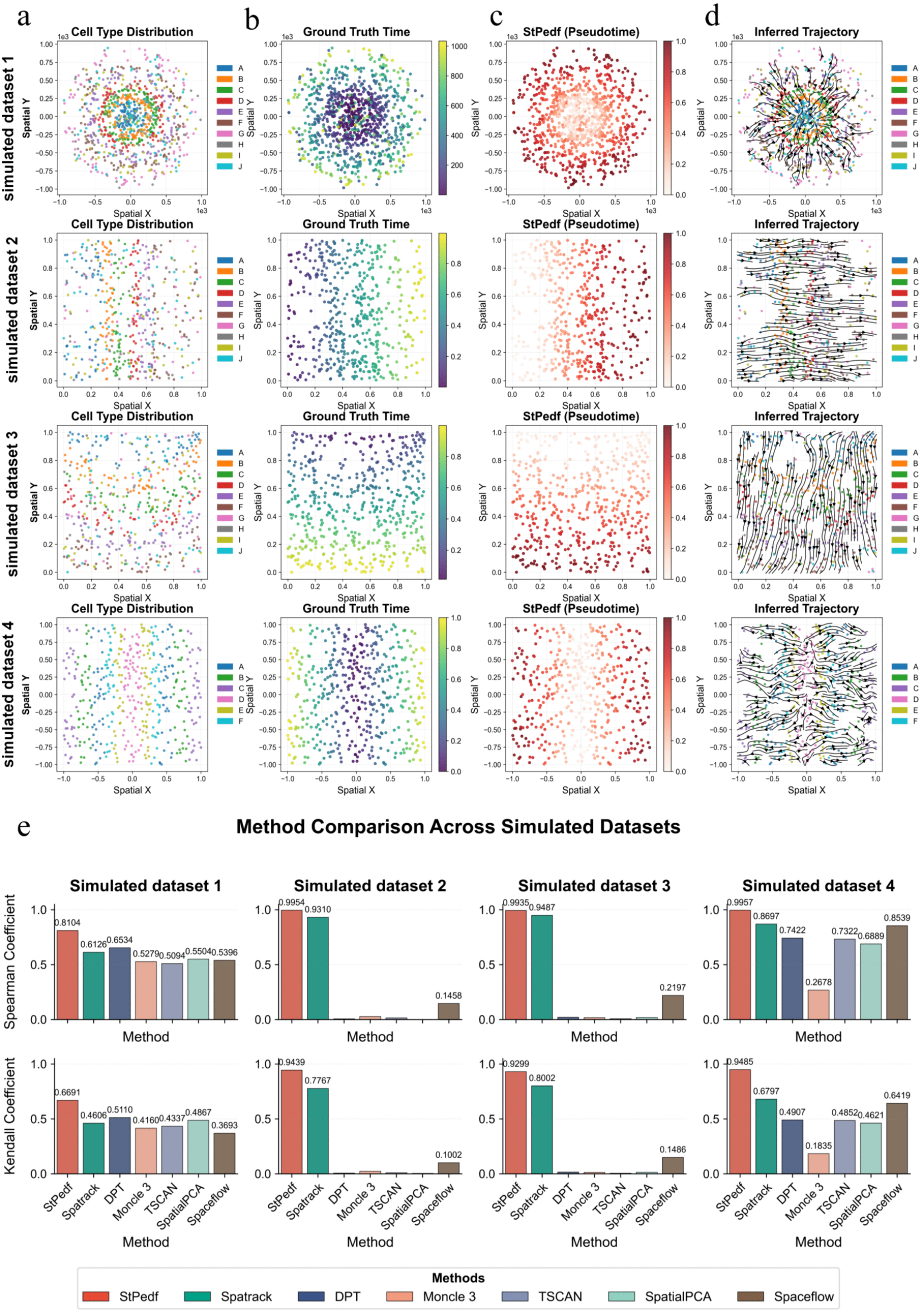
Benchmark analysis using four simulated datasets. **a.** Spatial distribution of cell types in the four simulated datasets. **b.** Spatial distribution of true values for the four simulated datasets. **c.** Pseudo-temporal-spatial distribution predicted by StPedf. **d.** Trajectories inferred by StPedf. **e.** Different methods applied to the group of simulated datasets, with the performance of each method evaluated using Spearman’s correlation and Kendall’s correlation between true values and the rankings of predictions made by each method.

To systematically evaluate the performance of StPedf, we construct five simulated datasets by superimposing spatial mapping patterns onto the dyngen simulator [28], with each dataset containing unspliced counts, spliced counts, and ground-truth developmental time information. Detailed parameter settings and the data-generation procedure are provided in the Supplementary Materials (S2 Table). Among these, simulated datasets 1–4 correspond to a bifurcating structure, a trifurcating structure with a linear spatial pattern, a trifurcating structure with a nonlinear spatial pattern, and an independent dual-path structure, respectively, whereas simulated dataset 5 further represents a multi-section, multi-timepoint scenario. The ground truth of the five basic simulated datasets (Sim1–Sim5) reflects clear developmental ordering, and StPedf effectively recovers the differentiation directions in these different topologies, accurately infers trajectory directionality, and reconstructs the pseudo-spatiotemporal map (Figs 2c and S1) and state-transition trend map (Figs 2d and S1).

Under the same experimental conditions, we further compare StPedf with DPT, Monocle3, TSCAN, SpaTrack, SpatialPCA, and SpaceFlow, and use Spearman and Kendall correlation coefficients to evaluate the consistency between inferred pseudotime and true developmental time. The results show that StPedf achieves the best correlation performance on Sim1–Sim4 (Fig 2e and S3 Table), indicating that it more accurately recovers the true developmental order under different topological structures. For the more challenging multi-section time-series simulated dataset Sim5, we further compare StPedf with SpaTrack. The results show that StPedf recovers a more continuous global pseudotime gradient and more coherent state-transition trends in the shared embedding space (S1 Fig), while the globally projected pseudotime distributions of different lineages also show clear separation (S2 Fig). Quantitative evaluation further shows that StPedf outperforms SpaTrack in both global ranking consistency and section-wise pseudotime recovery accuracy (S3 Fig), indicating that StPedf more accurately reconstructs cell ordering consistent with the true pseudotime in multi-section, multi-timepoint settings.

In addition, we perform stability analyses across different random seeds on all five simulated datasets. For each dataset, StPedf is run independently 30 times using different random seeds, and Spearman and Kendall correlation coefficients are calculated between the inferred pseudotime and the ground-truth time. The results show that, except for Sim1, the other datasets exhibit high consistency and relatively small variation across random seeds (S4 Fig), suggesting that StPedf has good robustness and reproducibility across different topological structures and multi-section scenarios.

To validate the advantages of the secondary utilization of spatial information, the spatial density adaptive module, and the joint embedding of gene and spatial information, this work constructs three variants of StPedf (S5 Fig): StPedf w/o Spatial Reuse that removes the secondary utilization of spatial information, StPedf w/o Adaptive Density that removes the spatial density adaptation mechanism, and StPedf w/o Embedding that uses only raw gene information without joint gene-space embedding. Ablation experiments are conducted on five simulated datasets. Compared with the three ablation variants, StPedf yields higher average Spearman correlation coefficients and average Kendall rank correlation coefficients in the comprehensive evaluation over the five datasets. These results clearly validate the advantages of the three components in StPedf.

We also compare StPedf with DPT on embedding and PAGA on embedding, both based on the same embeddings. Both comparison methods use features generated by the embedding module in our framework as input. Quantitative results (S6 Fig) show that StPedf achieves the highest Spearman correlation coefficients and Kendall’s tau-2 correlation coefficients, significantly outperforming the combinations of “DPT on embedding” and “PAGA on embedding” Qualitative analysis (S7 Fig) further corroborates this finding. Across four simulated datasets, the pseudo-time fields inferred by StPedf exhibit exceptionally high spatial continuity and smoothness, accurately reflecting developmental trajectories. In contrast, results from DPT or PAGA inference using the same embeddings display noticeable local irregular fluctuations and pseudo-time jumps. This intuitively demonstrates that OT inference within the StPedf framework more effectively leverages the fused features provided by the embedding module, transforming them into continuous trajectories that satisfy physical spatial constraints. Even starting from identical feature bases, the OT inferencer employed by StPedf significantly outperforms traditional inferencers like DPT and PAGA. This validates the unique advantages of OT as a spatial trajectory inferencer and demonstrates the effectiveness of the overall StPedf framework design.

### 2.3. Analysis of the trajectory and lineage dynamics of telencephalon regeneration in the Ambystoma mexicanum

We apply StPedf to an *Ambystoma mexicanum* telencephalon dataset, reconstructing the spatial trajectories of telencephalon regeneration following injury in *Ambystoma mexicanum*. Brain regeneration requires the coordinated activation of complex responses in a time- and region-specific manner. Spatial transcriptomics data highlight the spatial characteristics of cells in the telencephalon of *Ambystoma mexicanum* during developmental and injury-induced regeneration processes [29]. Further characterization of the activation and functional regulation of ependymal glial cells may provide new insights into enhancing brain regeneration in mammals. Based on previous studies, the regeneration process primarily occurs between 5 DPI and 20 DPI, so we select data from five time points (5, 10, 15, and 20 DPI) and use StPedf to construct detailed local trajectories of telencephalon regeneration in the *Ambystoma mexicanum*.

**Fig 3:**
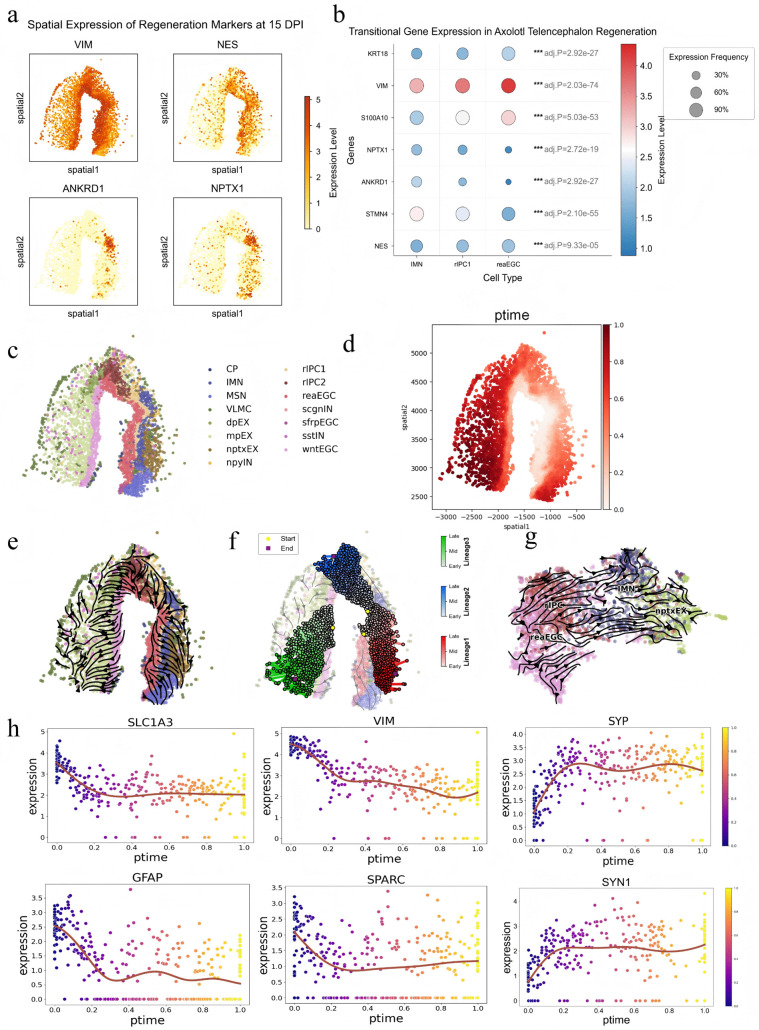
StPedf constructs the brain regeneration trajectory of *Ambystoma mexicanum.* **a.** A spatial visualization heatmap shows the expression patterns of key markers associated with cell regeneration in the injured region of the slice. **b.** A bubble plot reflects the expression dynamics of the reegc, rIPC1, and IMN marker genes, which are the primary cell types involved in *Ambystoma mexicanum* telencephalon regeneration. **c.** Spatial distribution of cell types during the 15th day regeneration phase. **d.** Pseudo-spatiotemporal plot of the 15th day regeneration phase following telencephalon injury in *Ambystoma mexicanum* **e.** Spatial trajectory of the 15th day regeneration phase following telencephalon injury in *Ambystoma mexicanum*. **f.** Shortest paths of lineages 1, 2, and 3 expanding in space during the 15th day regeneration phase following telencephalon injury in *Ambystoma mexicanum*. **g.** Complete regeneration trajectories obtained by integrating four samples. The UMAP plot shows a total of 2,880 cells. **h.** Trends in the pseudo-temporal dependence genes *SLC1A3*, *GFAP*, *VIM*, *SPARC*, *SYP*, and *SYN1* of lineage 1, selected by fitting a generalized additive model.

At 15 days post-injury (15 DPI) in the axolotl telencephalon regeneration sample, previous studies have shown that injury induces a continuous regenerative program involving reactive ependymoglial cells (reaEGCs), regeneration intermediate progenitor cells (rIPC1s), immature neurons (IMNs), and excitatory neuronal states including Nptx1-expressing excitatory neurons (nptxEXs) [29,30]. Based on this established biological background, we first illustrated the spatial expression patterns of several key regeneration-related marker genes in the tissue (Fig 3a), and further compared the dynamic expression of representative marker genes across different regeneration-related cell states (Fig 3b). A regeneration-specific transitional cell population was observed between reactive ependymoglial cells and immature neurons, corresponding to regeneration intermediate progenitor cells [31–32]. As shown in Fig 3b, this transitional population co-expressed marker genes associated with reactive ependymoglial cells (such as *VIM*, *NES*, *KRT18*, and *S100A10*) and marker genes associated with immature neurons (such as *ANKRD1*, *STMN4,* and *NPTX1*). These marker genes exhibited dynamic changes, reflecting the progression of cell states from injury response through progenitor transition to neuronal differentiation and maturation. Among them, *KRT18* expression gradually decreased, suggesting the loss of epithelial-like or progenitor features, whereas *VIM* expression also progressively declined, indicating the gradual attenuation of reactive glial and migration-related characteristics. Fig 3c shows the spatial distribution of different cell states in the tissue. On this basis, StPedf reconstructed the pseudo-spatiotemporal map of the 15 DPI regeneration sample (Fig 3d) as well as more detailed internal regenerative trajectories (Fig 3e). To further characterize the continuous transition paths of different regenerative branches, we applied the least action path (LAP) algorithm (S1 Note) to analyze three representative trajectories.

Lineage 1 corresponds to the regenerative pathway from the wound center to the right margin of the telencephalon, enriched with injury response pathways (such as glial cell activation-related pathways), progenitor cell proliferation and differentiation pathways, and neuronal maturation-related pathways. Gene expression changes over pseudo-time (Fig 3h and S2 Note) show that during the injury response phase dominated by reaEGCs, genes associated with glial activation, such as *SLC1A3* and *GFAP*, are highly expressed. During the progenitor cell transition phase, genes such as *VIM* and *SPARC* exhibit dynamic expression changes, aligning with the functional transition of rIPC1s from reaEGCs differentiation to neuronal maturation. During the neuronal maturation stage, synaptic-related genes such as *SYP* and *SYN1* are significantly upregulated, indicating the maturation of nptxEXs. Along the cellular differentiation axis, reaEGCs initiate repair, *rIPC1s* bridge the injury zone and the neuronal maturation zone, IMNs initiates electrophysiological function initialization, and nptxEXs completes functional reconstruction, with a clear hierarchical structure. This lineage reveals the core pathway from injury response to neuronal regeneration and functional recovery in the brain of the *Ambystoma mexicanum* following injury. The changes in *SLC1A3, VIM, GFAP,* and *SPARC* over pseudo-time as demonstrated by ISORT tend to rise more prominently in the middle to late stages (S9 Fig). While it exhibits some ability to distinguish between internal states within lineages, its differentiation between early initiation states and terminal maturation states, as well as the degree of recovery, is less clear than that of StPedf.

Lineage 2 and lineage 3 also exhibit functional specificity during telencephalon regeneration in *Ambystoma mexicanum*. Each lineage demonstrates distinct mechanisms of action, regulatory orientations, and adaptive environments (Figs 3f, S8**a**, and S8**b**). Lineage 2 corresponds to the dorsal lesion zone of the telencephalon and is the key lineage for dorsal neural circuit regeneration. It involves multiple overlapping pathways related to progenitor cell differentiation and neuronal subtype specialization, ensuring that specialized neurons adapt to the structural and functional demands of the dorsal telencephalon and the complex regenerative microenvironment. Lineage 3 corresponds to the medial non-injured region of the telencephalon, serving as the foundational lineage for maintaining normal telencephalic neural homeostasis. It is enriched with pathways related to normal development (e.g., neural developmental stemness maintenance pathways) and functional maintenance of mature neurons. Lineage 1 and 2 originate from injury-responsive subpopulations like reaEGCs, guided by signals from the wound surface and dorsal injury microenvironment to differentiate into functionally specialized regenerative lineages. Lineage 3 originates from wntEGCs, guided by the developmental microenvironment of the medial non-injured zone to maintain normal developmental trajectories. Lineage 1 drives neural circuit regeneration in specific regions, Lineage 2 adapts to the regenerative demands of complex dorsal areas, and Lineage 3 maintains normal brain region function. These three lineages synergistically propel the regenerative process from injury to partial functional recovery and overall homeostasis maintenance in the telencephalon, collectively supporting the orderly progression of the axolotl’s telencephalon regeneration.

To overcome the limitations of single-time-point spatial transcriptomics data in analyzing continuous evolution, we conduct multi-time-point analysis to validate the findings from the spatial analysis. We select spatial transcriptomics data from four time points—D5, D10, D15, and D20—following telencephalon injury in axolotls, covering the entire progression from “early injury response” to “regeneration peak” to “repair and remodeling.” StPedf independently calculates cell transition probabilities at each time point, using reactive ependymal glial cells (reaEGCs) and regenerative intermediate progenitor cells (rIPC1s) as starting points. By integrating all probability matrices, it overcomes the single-time constraint, combining spatial transcriptomic data from multiple time points (D5–D20) to construct a complete trajectory of telencephalon regeneration (Fig 3g). StPedf captures numerous intermediate cells like rIPC1s and IMNs. Their lineage transitions along pseudo-time further validate the reaEGC–rIPC–IMN–nptxEX model, demonstrating trajectory continuity. Compared to single-time-point analysis, the multi-time-slice integration yields more continuous trajectories, revealing the dynamic linkage mechanisms between injury response, progenitor cell proliferation, and neuronal functional maturation. It also highlights the core process whereby reaEGCs repair the wound through proliferation and differentiate into mature neurons. [30,33]

To systematically evaluate the ability of different methods to characterize the dynamic process of telencephalon regeneration in newts, we further compare the pseudo-spatiotemporal reconstruction performance of StPedf with that of Monocle3, Slingshot, SpaceFlow, and ISORT on this dataset (S10 Fig). Monocle3 exhibits substantial noise, whereas Slingshot captures a global trend, although this trend remains weak. Both Monocle3 and Slingshot fail to clearly connect progenitor-like states at the injury center with the outwardly expanding intermediate and mature states. SpaceFlow fails to reconstruct an effective pseudo-spatiotemporal structure in *Ambystoma mexicanum* data, with a severely constrained pseudotemporal dynamic range. Most cells are compressed into early states, and only a few discrete regions display localized terminal signals, resulting in a lack of interpretable spatial gradients. ISORT shows a certain degree of spatial stratification, but its trajectory directions contradict the known regeneration sequence. Trajectory streamline plots further reveal that ISORT produces disordered flow patterns with local reverse convergence, and reaEGC-associated regions are incorrectly identified as terminal states. In contrast, StPedf reconstructs a continuous, smooth, and directionally coherent pseudo-temporal gradient. Its streamline map displays more natural and coherent flow patterns, clearly depicting the progressive dynamic process from the regeneration initiation zone to the differentiation termination zone.

In summary, StPedf’s reconstructed dynamic model of telencephalon regeneration in the *Ambystoma mexicanum* systematically reveals the cross-scale regulatory mechanisms of “injury response-cell transformation-functional reconstruction” in neural regeneration, from molecular characteristics and lineage differentiation to spatiotemporal trajectories.

### 2.4. Characterization of tumor lineage differentiation and spatiotemporal heterogeneity

**Fig 4:**
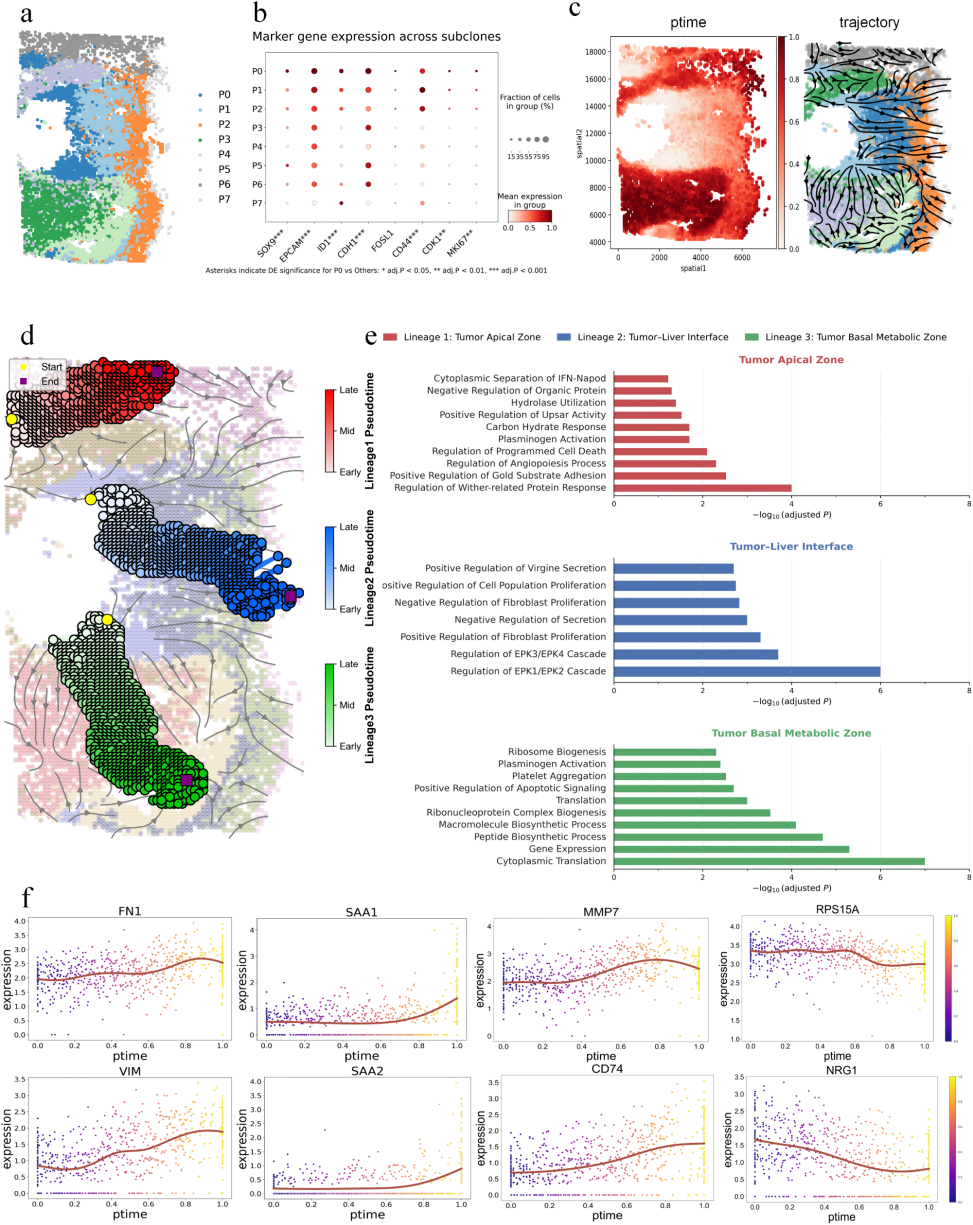
StPedf constructs the trajectory of tumor lineage differentiation. **a.** Spatial distribution of tumor subclones. **b.** Expression levels and proportions of target genes in each subclone. **c.** Pseudo-spatiotemporal map and spatial trajectory map of tumor expansion. **d.** The shortest paths of the three lineages in tumor spatial expansion, labeled in red, blue, and green, respectively. **e.** Functional annotation of pseudo-temporal dependent genes in the three lineages. **f.** Trends in gene expression changes.

The tumor microenvironment often contains more complex spatial structures and a higher degree of cellular heterogeneity. Tumor spatiotemporal heterogeneity profoundly affects tumor progression and therapeutic response, and represents a core challenge in precision medicine [34]. In this study, we use spatial transcriptomic data from primary ICC tumors and adopt the malignant subset classification identified by Wu et al. [35] based on expression profiles and spatial neighborhood information. Primary ICC tissue contains eight tumor subclones (P0–P7) with significant molecular differences (Fig 4a). These subclones show heterogeneous spatial distribution, among which boundary regions are enriched with features such as immunosuppressive signals and metabolic reprogramming, suggesting that clones undergo directional evolution along spatial directions. In the P0 subclone, multiple genes are coordinately highly expressed (Figs 4b and S11). For example, stemness markers (*SOX9*, *EPCAM*) cooperate with epithelial genes (*CDH1*) to maintain cellular stemness and epithelial structural properties. Meanwhile, active cell-cycle genes (*CDK1*, *MKI67*) in P0 drive rapid proliferation, indicating that the P0 subclone acts as tumor-initiating cells to drive heterogeneous evolution. We use StPedf to reconstruct cellular evolutionary trajectories starting from P0, identify three independent lineage origin patterns (Figs 4c, 4d, and S6**a**), and dissect the functional enrichment characteristics and spatial distribution rules of different tumor cell lineages, revealing the spatial differentiation logic of tumor progression.

The key challenge in the ICC dataset is not merely to recover a smooth pseudo-spatiotemporal map, but to reconstruct a spatiotemporal organizational pattern that is consistent with the tumor-initiating clone, spatial branch expansion, and boundary invasion programs. Comparison of the pseudotime visualizations (S12a Fig) generated by different algorithms shows that Monocle produces a highly noisy pseudotime distribution, with pronounced local interleaving of high- and low-value regions. Although Slingshot yields a smoother pattern than Monocle, it primarily reflects large-scale regional differences and fails to adequately capture the gradual transitions among neighboring clones within the tumor. SpaceFlow shows a severely restricted pseudotime dynamic range, with the vast majority of locations compressed into the initial stage and only a very small number of regions displaying localized high values, thus failing to form a continuous gradient. Although ISORT captures some degree of regional heterogeneity, it does not correctly identify the initiating cluster. In the comparison of trajectory streamline patterns (S12b Fig), ISORT generates relatively rich streamlines, but exhibits obvious local convergence and directional perturbation overall, with some trajectories ultimately pointing toward the central region. By contrast, StPedf not only accurately captures the pseudotemporal sequence from the early tumor center to the late tumor periphery, but also preserves the natural continuous transitions within regions and recovers the processes of multidirectional clonal expansion and boundary invasion.

To clarify the biological differences between different lineages, we use the LAP algorithm for inference, screened out trajectory-dependent genes along the optimal differentiation path, and perform pathway enrichment analysis on each lineage (Figs 4e and S13). Lineage 1 (P0-P5-P6) corresponds to the tumor apical expansion zone, enriched for cell-substrate adhesion regulation, apoptosis and death regulation, and NF-κB stress response pathways. It achieves local colonization through adhesion regulation and stress adaptation, does not participate in distant metastasis processes, and serves a spatial filling function. Lineage 2 (P0-P1-P2-P7) corresponds to the tumor-liver parenchyma boundary invasion zone, enriched in mitogen-activated protein kinase (*MAPK*) pathway regulation (such as *ERK1/ERK2* cascades, positive regulation of *MAPK* cascades), fibroblast proliferation regulation, and negative regulation of coagulation pathways, highly compatible with the invasive microenvironment at the tumor - liver parenchyma boundary invasion microenvironment, activating metastasis signals and remodeling the microenvironment, thereby becoming the core subgroup driving distant metastasis. Among the genes associated with lineage 2 (Fig 4f), EMT-related genes (e.g., *FN1*, *VIM*) exhibit synergistic fluctuations, aligning with the requirements of the tumor “breakthrough-colonization” stage and constituting the core molecular characteristics driving tumor boundary invasion. Immune suppression genes (such as *SAA1*, *SAA2* [36]) are highly expressed locally in the later stages, dynamically reflecting the construction process of the immune-suppressive microenvironment. Gradiently upregulated genes (such as *MMP7*, *CD74*) show progressive increases in expression over pseudo-time, dynamically presenting a series of processes involving the degradation of extracellular matrix components and the antagonism of the immune response against tumors. Basal maintenance genes (such as *RPS15A* and *NRG1*) are stably highly expressed, providing continuous energy support for invasion. The expression trends of these genes all reflect the role of lineage 2 in breaking through the tumor boundary and initiating metastasis. The genes *SAA1, SAA2*, and *CD74* recovered by ISORT primarily exhibited a decline with pseudo-time, while *MMP7* and *NRG1* showed weaker changes (S14 Fig). The gene expression patterns over pseudo-time reveal that ISORT is more sensitive to the transition from early-stage immune activity to late-stage attenuation within lineages. However, ISORT does not clearly reflect the late-stage enhancement of the tumor border invasion program as distinctly as StPedf. Lineage 3 (P0-P3-P4) corresponds to the metabolic zone at the bottom of the tumor, enriched with protein translation (such as cytoplasmic translation and gene expression), ribosomal biogenesis, and peptide/macromolecular biosynthesis pathways. By reinforcing anabolic metabolism to sustain core proliferation, it lacks active metastatic potential but provides metabolic support for tumor development.

All three lineages originate from tumor subclone P0, but diverge into functionally distinct subpopulations guided by microenvironmental signals including mechanical stress in the apical zone, immunosuppressive signals at the tumor margin, and metabolic stress in the basal zone. Lineage 1 fills the apical space to expand the tumor mass, Lineage 2 breaches the tumor boundary to initiate metastasis, and Lineage 3 enhances core metabolism to sustain proliferation. The synergistic evolution of these distinct lineages drives overall tumor expansion. Understanding the functions of each lineage facilitates targeted drug development to precisely strike different functional units within the tumor.

### 2.5. Reconstruction of the pseudo-spatiotemporal relationships and developmental trajectories of human DLPFC

In the human DLPFC sample 151673, we successfully reconstruct biologically plausible cortical developmental trajectories using StPedf. It is known that development begins in white matter (WM) and progresses through layers 6, 5, 4, 3, and 2 [14,37,38]. StPedf reveals pseudo-spatiotemporal relationships between cells, and the resulting pseudo-spatiotemporal map (pSM) differs significantly from traditional pseudo-temporal analysis, which focuses solely on intercellular expression similarity. The pSM integrates spatial positional relationships between cells with transcriptional expression patterns, overcoming the limitations of traditional methods that rely solely on transcriptional similarity. This provides a more comprehensive and biologically relevant analytical dimension for studying cortical developmental trajectories.

To investigate the biological functions associated with the pseudo-temporal developmental trajectories of the DLPFC, we further perform gene set enrichment analysis on genes showing significant changes along the pseudo-temporal sequence (S15 Fig). Multiple highly significant neurobiological functional enrichments are identified, including synaptic signaling, neurotransmitter transport, positive regulation of neuronal projection development, and regulation of NMDA receptor activity. The identified pseudotime-associated genes validate that our constructed pseudotime trajectories effectively capture key processes of neuronal functional maturation and synaptic assembly, while providing crucial molecular-level insights into cellular dynamics during cortical layer formation.

**Fig 5:**
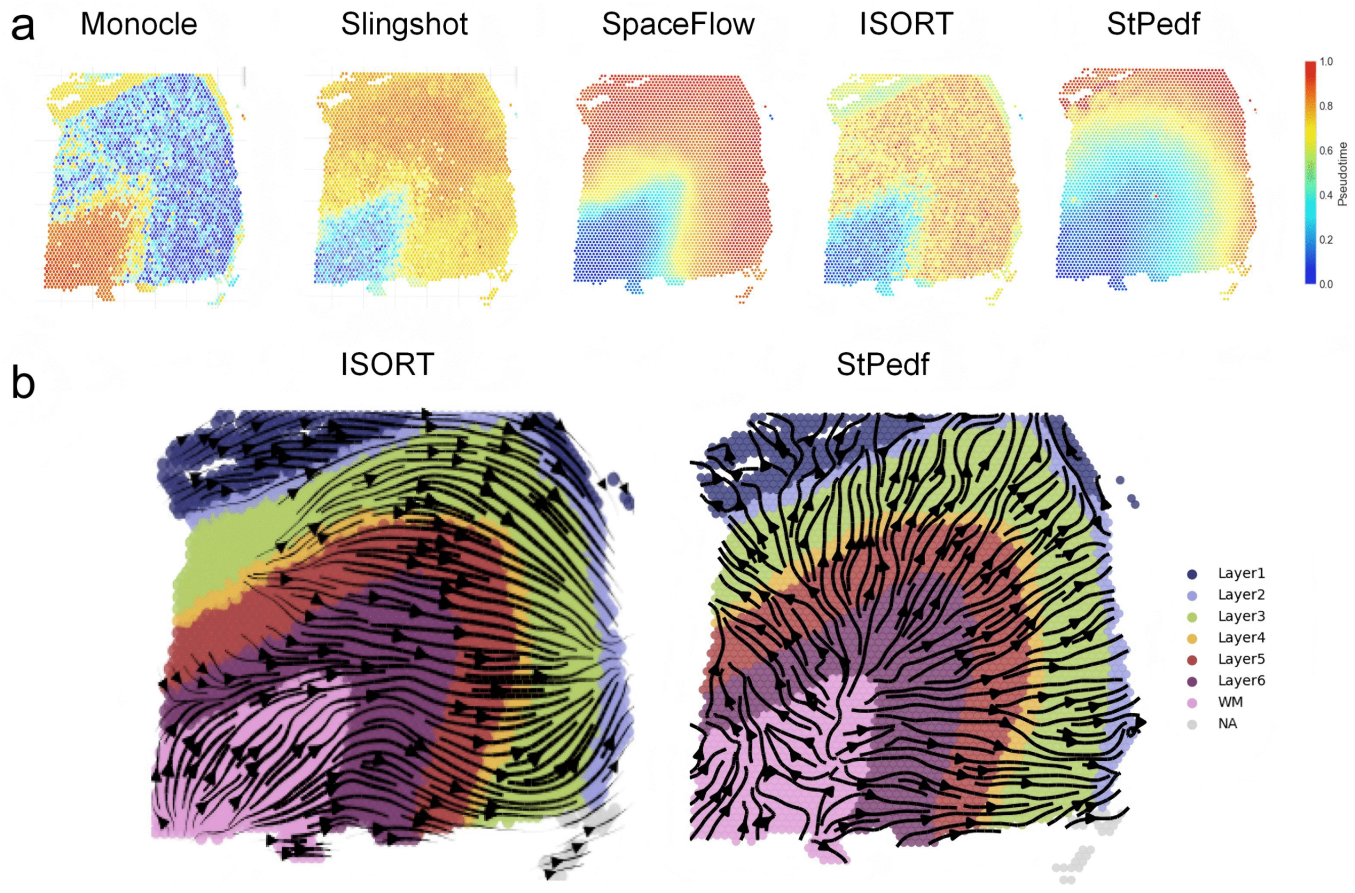
Comparison of StPedf with other DLPFC pseudo-spatiotemporal and trajectory analysis methods. a. Pseudo-spatiotemporal maps inferred by Monocle, Slingshot, SpaceFlow, ISORT, and StPedf. b. Inferred spatial trajectories of sample ID151673 inferred by ISORT and StPedf in physical space.

In the spatial visualization of pseudo-time generated by the traditional single-cell pseudo-time algorithms Monocle and Slingshot, we observe that the pseudo-spatiotemporal maps produced by Monocle and Slingshot lack hierarchical patterns and exhibit significant noise (Fig 5a). Furthermore, Monocle displays developmental trajectories from intermediate layers to WM layers and layer 1, which is inconsistent with the actual developmental sequence. In contrast, the three tested spatially-aware algorithms—SpaceFlow, ISORT [39], and StPedf—all produce layered pSM with clear, smooth color gradients (Fig 5a), indicating a pseudotemporal sequence from white matter (WM) to layer 1. This sequence reflects the correct in-to-out developmental order of the cortex, the layered spatial organization of the tissue, and underscores the importance of spatial information. Among these, SpaceFlow exhibits distinct and smooth color gradients, yet it has limitations in maintaining consistency of spatial structures both within and across cortical layers. ISORT gradients are less smooth and exhibit noticeable noise. StPedf more precisely corresponds to the layered spatial organization, better preserving spatial structures both within and across cortical layers. This enables clearer interpretation of the relationship between spatial structure and biological stratification. Compared to SpaceFlow and ISORT, StPedf offers superior advantages in reconstructing authentic tissue spatial stratification, developmental associations, and gradient smoothness. Regarding cell velocity comparisons (Fig 5b), both SpaRNA velocity from ISORT and StPedf permit visualization of detailed intra-layer growth. However, ISORT exhibits cross-layer reverse trajectories—specifically, arrows pointing from Layer 3 toward deeper regions like Layer 5/6—alongside disordered trajectories lacking stratification associations. manifested as arrows randomly traversing within Layer 4. In contrast, StPedf displayed correct growth trajectories from WM to sequence layers.

It is important to emphasize that only StPedf can more closely match the actual layering of the cortex, demonstrating a smooth progression between adjacent layers and a higher degree of color gradation that matches the spatial organization of cortical development. At the same time, the speed obtained reconstructs the correct growth trajectory from WM to the sequence layer, visualizing the detailed internal growth within the layer.

## 3. Discussion

In this work, we propose StPedf, which encodes spatial transcriptomic data into low-dimensional embeddings via deep learning. It adaptively fuses these embeddings with spatial information through density-based spatial integration, leveraging spatial guidance to derive cell-to-cell transition matrices through optimal transport for trajectory inference. By integrating graph neural networks with convolutional neural networks, the model naturally captures the complex nonlinear interactions between high-dimensional gene expression and spatial topology, reconstructing continuous evolutionary pathways of cellular states. Pseudo-spatiotemporal graphs reveal spatiotemporal relationships among cells or points within spatial transcriptomics data, while velocity visualization details internal growth dynamics. Cell trajectories across temporal samples can be directly tracked via OT mapping.

To evaluate the performance of StPedf, we test it on five simulated datasets (Figs 2 and S3). Sim1–Sim4 are used for benchmarking against other mainstream trajectory inference methods, including SpaTrack, DPT, Monocle3, TSCAN, SpatialPCA, and Spaceflow. Sim5 is used for a direct comparison with SpaTrack. The results demonstrate that StPedf achieves superior performance in all comparisons. Furthermore, we conduct ablation studies on all five simulated datasets using three variants: without Spatial Reuse, without Adaptive Density, and without Embedding. The results further confirm the advantages of StPedf. The results of these experiments (S5 Fig) show that incorporating spatial reuse, adaptive density utilization, and joint gene-space embedding improves the accuracy of trajectory inference while adaptively balancing the contributions of spatial and gene information, further confirming StPedf’s advantages. We further assess StPedf’s performance on three key spatial transcriptomics datasets from different species and biological contexts: brain regeneration in the *Ambystoma mexicanum*, tumor expansion and metastasis, and human DLPFC development. In *Ambystoma mexicanum* dataset, StPedf accurately reconstructs the regeneration dynamics model of the telencephalon at both single and multiple time points (Fig 3e and 3g). In the tumor dataset, StPedf successfully reconstructs cellular spatial trajectories starting from point P0 and identifies three distinct differentiation patterns with independent lineage origins (Fig 4c and 4d). In the DLPFC dataset, comparative analysis shows that mainstream methods such as Monocle, Slingshot, and Spaceflow fail to construct meaningful cell velocity fields. While ISORT produces trajectories, they appear disorganized and lack hierarchical relationships. Only StPedf generates trajectories that closely correspond to the actual cortical layering structure. It not only reveals smooth transitions between adjacent cortical layers but also shows strong alignment between trajectory color gradients and the spatial organization of cortical development (Fig 5a). Furthermore, StPedf accurately reconstructs developmental trajectories from white matter (WM) through successive cortical layers and visualizes detailed growth dynamics within each layer (Fig 5b). To further support the rationale of the original adaptive explicit spatial fusion module, we perform ablation experiments on both the *Ambystoma mexicanum* telencephalon regeneration dataset and the ICC tumor dataset, in which the adaptive explicit spatial fusion module is removed from the transition construction while the embedding term is kept unchanged. The results show that, after removing this module, the global state-transition trend plots display disordered local flow-field directions and reduced coherence (S17**a and** S17c Fig), while the pseudotime distributions become noticeably noisier, with fragmented local trajectories and reduced overall continuity (S17b and S17d Fig). In contrast, the full model recovers smoother and more coherent local flow fields and pseudotime gradients (Figs 3 **and** 4). These results indicate that the role of the adaptive explicit spatial fusion module is not to impose biological importance on high- or low-density regions, but rather to robustly balance spatial constraints and expression differences, thereby substantially improving the local continuity and overall robustness of trajectory inference in real tissues.

StPedf has demonstrated strong capability for spatial trajectory inference across complex biological contexts, providing a new computational framework for characterizing the spatial features of cell-state evolution. However, the current study still has several aspects that warrant further improvement. First, StPedf does not yet incorporate prior information such as cell–cell communication networks and dynamic regulatory cues into transition matrix construction, which limits its ability to characterize the relationship between microenvironmental signals and cell-state transitions. Second, although StPedf supports joint multi-slice analysis based on a shared embedding space, the current framework still relies on known temporal ordering or prior alignment information, and has not yet achieved automatic alignment of multiple slices or unified spatiotemporal trajectory inference. With the integration of more prior biological knowledge and further advances in multi-slice spatiotemporal modeling, StPedf is expected to enable more precise and systematic analysis of cell-fate dynamics in complex tissues.

## 4. Conclusion

In summary, StPedf effectively integrates high-dimensional gene expression with spatial topology information, fully leveraging spatial data to precisely reveal the continuous spatiotemporal evolution of cellular states. It demonstrates robust and unified analytical capabilities across diverse complex biological processes, including regeneration, development, and disease. It provides a powerful computational tool for understanding the spatial regulation of gene expression and cellular fate transitions, excels in cross-species and cross-scenario analyses, and offers critical technical support for theoretical life science research and clinical medical applications—such as tumor metastasis prediction, regeneration mechanism analysis, and brain development studies.

## 5. Methods

### 5.1. Spatial map construction based on Alpha Complex

In order to capture the spatial adjacency relationships between cells or spots, we construct a graph structure from the original spatial coordinate information:


$$\begin{array}{c} G=(V,E), \end{array}$$ (1)

where the node set V represents all cells/spots, with each node accompanied by its corresponding gene expression vector, the edge set E describes the spatial adjacency relationship between nodes. Next, we use the Alpha Complex method [40] to construct a Voronoi diagram and extract its one-dimensional skeleton as the adjacency edge set E. For each position $r\in {\mathbb{R}}^{\text{2}}$, its Voronoi cell is defined as:


$$\begin{array}{c} V(r)=\{x\in R^{\text{2}}|∥x-r∥\leq ∥x-r^{\prime }∥,\forall r^{\prime }\in C\}, \end{array}$$ (2)

where C is the set of coordinates for all points, and ‖⬝‖ denotes the Euclidean distance. Next, use the one-dimensional skeleton of Alpha Complex to determine the adjacency relationship:


$$\begin{array}{c} E=\left\{(i,j)|\underset{k\in \left\{i,j\right\}}{\cap }(V(r_{k})\cap B(r_{k},\delta ))=\emptyset \right\}, \end{array}$$ (3)

where B(x,δ) is a two-dimensional circular disk region with a center of x and a radius of δ. Take the average distance of the k nearest neighbors for each point. Then, the final adjacency matrix $A\in {\left\{\text{0},\text{1}\right\}}^{n\times n}$ is defined as follows:


$$\begin{array}{c} A_{ij}=\left\{ \begin{array}{cc} & \text{1},(i,j)\in E\\ & \text{0},otherwise \end{array}\right.\;. \end{array}$$ (4)

Since most nodes are not adjacent to each other, the adjacency matrix is usually sparse. Therefore, we use sparse matrix storage to save memory and computing resources. Data preprocessing is described in S3 **Note**.

### 5.2 Mask input generation

We mask the original gene expression matrix. Let the original expression matrix be $X\in {\mathbb{R}}^{n\times m}$, where n denotes the number of cells/spots and m denotes the gene dimension. We randomly select nodes from V to form a subset  U⊂V and mask the expression vectors of these nodes, replacing them with learnable vectors ${\tilde{x}}_{i}\;\in {\mathbb{R}}^{m}$. The masked expression matrix is defined as follows:


$$\begin{array}{c} X^{\prime }=\left\{ \begin{array}{cc} & {\tilde{x}}_{i},i\in U\\ & x_{i},i\notin U \end{array}\right.\;. \end{array}$$ (5)

The definition mask reconstruction loss is as follows:


$$\begin{array}{c} L_{mask}=\sum_{i\in M}\overset{\text{2}}{\underset{F}{\|x_{i}-{\tilde{x}}_{i}\|}}. \end{array}$$ (6)

### 5.3 Joint encoding of feature embedding and graph embedding

Using a dual-path encoding framework, gene expression features and spatial topological structures are processed independently, and features are fused in the latent space. The feature embedding encoder consists of two fully connected layers, which map the masked expression matrix $X\prime \in {\mathbb{R}}^{n\times m}$ to a low-dimensional feature representation $Z_{f}\;\in {\mathbb{R}}^{n\times h_{f}}$, i.e.,:


$$\begin{array}{c} H_{\text{1}}=ReLU(X^{\prime }W_{\text{1}}+b_{\text{1}}),W_{\text{1}}\in R^{m\times h},b_{\text{1}}\in R^{\text{1}\times h}, \end{array}$$ (7)

$$\begin{array}{c} Z_{f}=f_{enc}(X^{\prime })=ReLU(H_{\text{1}}W_{\text{2}}+b_{\text{2}}),W_{\text{2}}\in R^{h\times h_{f}},b_{\text{2}}\in R^{\text{1}\times h_{f}}, \end{array}$$ (8)

where $W_{\text{1}}\in {\mathbb{R}}^{m\times h},W_{\text{2}}\in {\mathbb{R}}^{h\times h_{f}}$ are learnable parameters, and the output $Z_{f}\;\in {\mathbb{R}}^{n\times h_{f}}$ is the feature embedding of gene expression. This design uses a low-dimensional representation learning ($h_{f}\ll m$) model to mine gene co-expression patterns, forcing the model to learn gene co-expression patterns, while ReLU activation ensures feature non-negativity.

The graph embedding encoder uses a two-layer graph convolutional network (GCN) with the spatial adjacency matrix A and feature representation $Z_{f}$ as inputs, and outputs a space-aware graph embedding $\;Z_{g}\;\in {\mathbb{R}}^{n\times h_{g}}$. The normalization matrix is constructed as follows:


$$\begin{array}{c} \tilde{A}=A+I_{n}, \end{array}$$ (9)

$$\begin{array}{c} {\tilde{D}}_{ii}=\sum_{j}{\tilde{A}}_{ij}, \end{array}$$ (10)

where $\tilde{A}=A+I$ is the adjacency matrix after adding self-loops, $\tilde{D}$ is its degree matrix, and the probability parameters are generated after obtaining the normalized matrix:


$$\begin{array}{c} H^{\left(\text{1}\right)}=\text{ReLU}\left({\tilde{D}}^{-\frac{\text{1}}{\text{2}}}\tilde{A}{\tilde{D}}^{-\frac{\text{1}}{\text{2}}}Z_{f}W_{\text{0}}\right), \end{array}$$ (11)

$$\begin{array}{c} \mu ={\tilde{D}}^{-\frac{\text{1}}{\text{2}}}\tilde{A}{\tilde{D}}^{-\frac{\text{1}}{\text{2}}}H^{\left(\text{1}\right)}W_{\mu }, \end{array}$$ (12)

$$\begin{array}{c} og\sigma ={\tilde{D}}^{-\frac{\text{1}}{\text{2}}}\tilde{A}{\tilde{D}}^{-\frac{\text{1}}{\text{2}}}H^{\left(\text{1}\right)}W_{\sigma }, \end{array}$$ (13)

where $W_{\text{0}}\in {\mathbb{R}}^{h_{f}\times h_{g}},W_{\mu },W_{\sigma }\in {\mathbb{R}}^{h_{g}\times h_{g}}$, specifically, the operation form of the first layer GCN is:


$$\begin{array}{c} H^{\left(l+\text{1}\right)}=\sigma \left({\tilde{D}}^{-\frac{\text{1}}{\text{2}}}\tilde{A}{\tilde{D}}^{-\frac{\text{1}}{\text{2}}}H^{\left(l\right)}W^{\left(l\right)}\right), \end{array}$$ (14)

where σ(·) is the activation function and $W^{\left(l\right)}$ is the learnable parameter. Next, mean field probability modeling is performed, assuming that the output of the last layer is a graph embedding $Z_{g}$, which follows an isotropic Gaussian distribution:


$$\begin{array}{c} p(Z_{g}|A,Z_{f})=i=\prod^{\underset{n}{i=\text{1}}}N\left(\mu_{i},diag\left({\sigma_{i}}^{\text{2}}\right)\right), \end{array}$$ (15)

next, we perform differentiable sampling:


$$\begin{array}{c} z_{i}=\mu_{i}+\epsilon ⊙\sigma_{i},\epsilon \sim N(\text{0},I). \end{array}$$ (16)

The output $Z_{g}\in {\mathbb{R}}^{n\times h_{g}}$ is a spatial perception embedding, and its variance term $\;\sigma_{i}$ quantifies positional uncertainty. Finally, gene expression features and spatial topological information are fused through a concatenation operation:


$$\begin{array}{c} Z=[Z_{f}∥Z_{g}]\in R^{n\times (h_{f}+h_{g})}, \end{array}$$ (17)

where $h_{f}$ represents the gene feature embedding dimension, $h_{g}$ represents the spatial embedding dimension, and ∥ represents matrix row concatenation. This joint embedding retains both gene expression characteristics and spatial topological constraints, providing downstream tasks with information-rich representations. In the implementation of joint feature embedding and graph embedding, the random seed is fixed to 2025 to ensure the determinism of model weight initialization and subsequent training.

### 5.4. Multi-task training objectives

The training consists of two stages. The first stage is pre-training, where the model learns general embeddings by minimizing the following loss function:


$$\begin{array}{c} L_{pretrain}=\lambda_{rec}L_{rec}+\lambda_{gcn}L_{gcn}+\lambda_{self}L_{self}, \end{array}$$ (18)

where $\lambda_{rec}\;=10,\lambda_{gcn}=0.1,\lambda_{cluster}=1$ represents the fixed weighting for each type of loss.

The second stage optimizes the embedding space based on pretraining to enhance clustering performance. First, K-means initializes cluster centers on the pretrained embeddings, then minimizes the following loss:


$$\begin{array}{c} L_{fine-tune}=\lambda_{rec}\cdot L_{rec}+\lambda_{gcn}\cdot L_{gcn}+\lambda_{cluster}\cdot L_{cluster}, \end{array}$$ (19)

where $\lambda_{rec}\;=10,\lambda_{gcn}=0.1,\lambda_{cluster}=1$ represents the fixed weighting for each type of loss. At this stage, the mask reconstruction loss $L_{self\;}$ was removed to avoid conflict with the clustering objective, and a depth clustering loss was introduced. The clustering loss $\left({ℒ}_{\text{cluster}}\right)$ serves as an auxiliary regularization term. It is used to further stabilize the local structure, improve the robustness of embedding representations, and enhance the stability of downstream trajectory inference based on the pre-trained embeddings. After removing the clustering loss (S18 Fig), both the Spearman correlation coefficients (S18a Fig) and Kendall correlation coefficients (S18b Fig) between the model predictions and the ground-truth time labels decreased across the simulated datasets. For detailed information on the two-stage loss function, please refer to S4 **Note**.

### 5.5. Cell density-aware adaptive weighting mechanism and low-confidence region protection

To quantify the local cell distribution density, spatial density is introduced, and a continuous density field is constructed using Gaussian kernel density estimation. For any cell i, its density estimate ${\hat{\rho }}_{i}$ is defined as:


$$\begin{array}{c} {\hat{\rho }}_{i}=exp\left(\frac{\text{1}}{nb}\sum_{j=\text{1}}^{n}K\left(\frac{\left\|s_{i}-s_{j}\right\|}{b}\right)\right), \end{array}$$ (20)

where ${𝐬}_{i}\;\in {\mathbb{R}}^{\text{2}}$ is the spatial coordinate of the cell (typically the two-dimensional position of the tissue section), $K(u)=\frac{\text{1}}{\sqrt{\text{2}\pi }}e^{-u^{\text{2}}/\text{2}}$ standardized Gaussian kernel function, b is the kernel function bandwidth parameter (default value), which controls the spatial resolution of density estimation. This equation performs a weighted sum of n cells surrounding the cell  i  using the Gaussian kernel, then generates the density value ${\hat{\rho }}_{i}$ through an exponential transformation. This equation enhances the numerical contrast between high- and low-density regions while compressing the dynamic range of the original summation result to prevent numerical overflow. To eliminate the interference of global density differences between different tissue samples, ${\hat{\rho }}_{i}$ is normalized using Min-Max normalization:


$$\begin{array}{c} \rho_{i}=\frac{{\hat{\rho }}_{i}-{min}_{k}{\hat{\rho }}_{k}}{{max}_{k}{\hat{\rho }}_{k}-{min}_{k}{\hat{\rho }}_{k}+\varepsilon }, \end{array}$$ (21)

where $\varepsilon =10^{-10}$ is a smoothing parameter that ensures the denominator is not zero. After normalization, we obtain $\rho_{i}\in \left[0,1\right]$, where $\rho_{i}=1$ corresponds to the cell with the highest local density in the sample, and $\rho_{i}=0$ corresponds to the cell with the lowest density. While retaining the relative density ranking, the values are strictly constrained to a fixed interval, providing standardized input for subsequent weight mapping. To achieve adaptive correlation between density and weight, a reverse correlation function is designed to map normalized density $\rho_{i}$ to spatial weight $\alpha_{i}$:


$$\begin{array}{c} \alpha_{i}={\alpha iminmax}_{min} \end{array}$$ (22)

the lower bound $\alpha_{min}$ ensures that even in the highest-density regions $\rho_{i}\to 1$, spatial information is not completely ignored, thereby preventing the joint embedding from becoming overly dominant. The upper bound $\alpha_{max}$, in turn, ensures that in low-density regions $\rho_{i}\to 0$, spatial proximity can become the dominant factor. When $\rho_{i}\to 1,1-\rho_{i}\to 0$, and thus $\alpha_{i}\to \alpha_{min}$, so the transition matrix is mainly governed by similarity in the joint embedding space. Conversely, when $\rho_{i}\to 0$, $\alpha_{i}\to \alpha_{max}$, and the transition matrix is mainly governed by spatial proximity. This adaptive mechanism flexibly balances gene expression and spatial information according to the local cellular environment, while the dataset-specific choice of $\left(\alpha_{min},\alpha_{max}\right)$ accounts for variations in tissue architecture, density distribution, and trajectory complexity across different experiments. In high-density regions, many cells are already geometrically close to each other, and the original Euclidean distance is often highly redundant. Reducing the contribution of $d_{s}\left(i,j\right)$ at this stage avoids over‑strengthening local geometric proximity, thereby maintaining sensitivity to weak but potentially important expression differences. In low-density regions, spatial separation is more discriminative. Appropriately strengthening spatial constraints helps prevent unreasonable connections between cells that are transcriptionally similar but spatially disconnected. Detailed parameter descriptions can be found in S5 Note.

Low density does not always represent a biological signal, it can also arise from technical artifacts such as tissue edges, section tearing, hole-like sparse regions, or areas with poor local connectivity. To address this, we introduce a low-confidence region protection mechanism. We automatically identify spots potentially affected by technical factors using three types of local geometric and neighborhood statistical criteria: spots located at tissue edges, spots in hole-like sparse neighborhoods, and spots with unreliable local connectivity. For these low-confidence regions, we no longer apply the density-adaptive rule; instead, we directly assign a fixed conservative weight $\alpha_{prot}$ to prevent unreliable local density from dominating trajectory construction. For normal regions, spatial weights are still dynamically adjusted in the density-adaptive manner described above. The final weight ${\tilde{\alpha }}_{i}$ is expressed as:


$$\begin{array}{c} {\tilde{\alpha }}_{i}=\left\{ \begin{array}{cc} & \alpha_{prot},ifi\in low-confidenceregion\\ & \alpha_{i},otherwise \end{array}\right.\;. \end{array}$$ (23)

### 5.6. Adaptive fusion based on joint embedding and spatial prior knowledge

We separately compute the spatial distance between cells, denoted as $d_{s}\left(i,j\right)$, and the Euclidean distance derived from the joint embedding of gene expression and spatial information, denoted as $\;d_{g}\left(i,j\right)$. To ensure the comparability of these distance metrics, maximum normalization is applied to eliminate scale differences:


$$\begin{array}{c} d_{s}\left(i,j\right)=\frac{∥s_{i}-s_{j}{∥}_{\text{2}}}{{max}_{k,l}∥s_{k}-s_{l}{∥}_{\text{2}}}, \end{array}$$ (24)

$$\begin{array}{c} d_{g}\left(i,j\right)=\frac{∥z_{i}-z_{j}{∥}_{\text{2}}}{{max}_{k,l}∥z_{k}-z_{l}{∥}_{\text{2}}}, \end{array}$$ (25)

where $s_{i\;}$ denotes the coordinate vector of cell i in the original space, and $z_{i}\in {\mathbb{R}}^{d}$ denotes the joint embedding vector of cell i.It should be emphasized that spatial information enters the model not only through the intercellular spatial distance$d_{s}\left(i,j\right)$, the upstream spatial graph construction and joint embedding learning term $d_{g}\left(i,j\right)$ have already encoded the spatial structure. To effectively balance the spatial position constraints and the embedding information representation pattern, and to fully account for the non-uniformity of cell spatial distribution, we adjust the contribution of the explicit original spatial distance term and construct the comprehensive distance matrix M:


$$\begin{array}{c} M_{ij}={\tilde{\alpha }}_{i}\cdot d_{s}(i,j)+(\text{1}-{\tilde{\alpha }}_{i})\cdot d_{g}(i,j), \end{array}$$ (26)

this formula uses the local density weight $\alpha_{i}$ of cells i as a regulating factor to dynamically allocate the contributions of spatial distance and gene expression distance. In high-density regions ${\tilde{\alpha }}_{i}\to \alpha_{min}$: $d_{g}(i,j)$ becomes the dominant factor, giving priority to retaining the joint embedding pattern; in low-density regions ${\tilde{\alpha }}_{i}\to \alpha_{max}$: $d_{s}(i,j)$ becomes the dominant factor, giving priority to maintaining spatial topological consistency. In the comprehensive distance matrix  M, joint embedding $z_{i}$ provides advanced feature representation, while the original spatial coordinates provide key spatial structure a priori information for trajectory inference.

### 5.7. Generation of optimal transport transition probabilities based on entropy regularization

To quantify the possibility of cell-cell interactions, the Sinkhorn algorithm is used to solve the entropy-regularized optimal transport problem and construct the probability transition matrix P, whose expression is:


$$\begin{array}{c} P=argmin_{P\in \overset{n\times n}{\underset{+}{R}}}\left\{\langle P,M\rangle_{F}+\gamma \sum_{i,j}P_{ij}\;{log\;P}_{ij}\right\}, \end{array}$$ (27)

constraints:


$$\begin{array}{c} P\text{1}=a,\;P^{T}\text{1}=b,\;a=b=\frac{\text{1}}{n}\text{1}, \end{array}$$

where $\langle P,M\rangle_{F}=\sum_{i,j}P_{ij}M_{ij}$ is the Frobenius inner product, which measures the matching cost between transition probabilities and composite distances, γ=0.1 (default value) is the entropy regularization coefficient, which controls the smoothness of the solution. The larger γ, the closer P is to a uniform distribution. a,b is the marginal distribution vector, here set to a uniform distribution $a=b=\frac{\text{1}}{n}\text{1}$, ensuring that the sum of the out-degree and in-degree of each cell is equal (consistent with the physical meaning that “the sum of transition probabilities from each cell to other cells is 1/n”). In the generated P matrix, the element $P_{ij}$ represents the probability of functional association transition from cell  i  to cell j. High-probability transfer ($P_{ij}$≫0) corresponds to $\;M_{ij}$ smaller cell pairs, i.e., cells with similar gene expression and spatial proximity; low-probability transfer ($P_{ij}$ ≈0) corresponds to $\;M_{ij}$ larger cell pairs, such as cell pairs with significant gene phenotype differences or spatially isolated cells.

To prevent cells from transferring all probability mass to themselves and to avoid the emergence of a trivial solution, we set the diagonal elements of the constructed fused distance matrix $\;M_{ij}$ to a sufficiently large constant $d_{diag}=10^{\text{6}}$. Since both the spatial distance and gene expression distance have been normalized to the range [0,1], this value is significantly larger than all off-diagonal elements, effectively suppressing self-transition during the optimal transport solution and ensuring that the transition matrix P reflects genuine intercellular relationships. Sensitivity analysis indicates that when $d_{diag}=10^{\text{6}}$, the transport matrix is insensitive to the specific value (S16 Fig).

### 5.8. Selection of starting cells

The StPedf method provides two strategies for defining initial cells based on prior knowledge: one is a selection method based on cell type, which specifies a specific cell type as the starting point for development.


$$\begin{array}{c} S_{type}=\{i|c_{i}=C\}, \end{array}$$ (28)

where $c_{i}$ indicates the category label of cell i, where C is the target cell type. The second method is based on spatial coordinates and is suitable for cases where the location of the morphogenesis center is known. It uses the K-nearest neighbor algorithm to select neighboring cells:


$$\begin{array}{c} S_{\text{coord}}=\text{KNN}(s_{c},k), \end{array}$$ (29)

where $s_{c}\;=\;\left(x_{c},\;y_{c}\right)$ is the morphogenesis center coordinate, and k is the parameter for the number of neighboring cells. After determining the initial cell set S, the algorithm constructs a transition probability matrix through an optimal transport model and calculates the cell development sequence using the probability accumulation method:


$$\begin{array}{c} O_{i}=\sum_{j\in S}P_{ji}, \end{array}$$ (30)

where $P_{ji}$ represents the transfer probability from the starting cell j to the target cell i. Based on this probability distribution, the cells are sorted in ascending order.


$$\begin{array}{c} r_{i}=rank\left(O_{i}\right)-\text{1}, \end{array}$$ (31)

where $rank\left(O_{i}\right)$ represents $O_{i}\;\;$ in ascending order among all cells. Finally, it is normalized to a pseudo-time parameter:


$$\begin{array}{c} \tau_{i}=\frac{r_{i}}{N-\text{1}}, \end{array}$$ (32)

where N is the total number of cells, $\tau_{i}\in \left[0,1\right]$. Biologically, $\tau_{i}\approx 0$ corresponds to the initial stage of development (high differentiation potential), $\tau_{i}\approx 1$ corresponds to the final stage of differentiation, and the intermediate value reflects the continuous transition stages of development.

### 5.9. Trajectory Inference strategy for multi-timepoint / section spatial transcriptomic data

Since a single timepoint or section is often insufficient to fully capture all cell states in a temporal biological process, joint analysis of multiple timepoints or sections can cover intermediate transitional states over a wider range and thus reconstruct continuous biological processes more completely. For this reason, we adopt an analytical strategy of “separate inference per sample, joint interpretation in a shared embedding space” for multi-timepoint and multi-section spatial transcriptomic data. First, cells from all timepoints or sections are concatenated row-wise to form a unified data object, while retaining the original spatial coordinates, time labels, and cell type annotations of each cell. On this basis, principal component analysis is performed using highly variable genes (with timepoints used as batch information during selection), followed by UMAP embedding to obtain a shared low-dimensional representation for all cells. This embedding space maps cells from different timepoints or sections into the same coordinate system, eliminating batch differences between samples while preserving the main biological continuity.

After the shared embedding space is constructed, the data is split into independent subsets by timepoint or section, and the corresponding coordinates of cells in the shared embedding space are extracted for each subset based on cell indices. For each subset, a starting cell state is first specified according to known biological background or prior knowledge, and StPedf is used within the subset to construct a cell–cell transition probability matrix to characterize local potential transition relationships. Pseudotime and velocity fields are then calculated based on this transition matrix. After trajectory inference is completed independently within each subset, the low-dimensional coordinates and velocity vectors inferred from different timepoints or sections are aggregated into the shared embedding space, and the overall trajectory trend is visualized through velocity field interpolation and streamline plotting on a unified grid.

### 5.10. Cell velocity calculation and trajectory construction methods

First, to accurately calculate cell migration speed, this study proposes a dual-modality neighbor selection strategy that integrates both spatial proximity and functional similarity. Specifically, for each cell i, its neighbor set is defined as the union of spatial neighbors and embedded neighbors:


$$\begin{array}{c} N_{i}={N_{i}}^{spatial}\cup {N_{i}}^{embed}, \end{array}$$ (33)

where ${N_{i}}^{spatial}$ represents the K-nearest neighbor set based on spatial coordinates (of size $k_{p}$), and ${N_{i}}^{embed}$ represents the K-nearest neighbor set based on joint embedding (of size $k_{e}$). This mechanism emphasizes spatial continuity ($k_{p}\gg k_{e}\;$) in spatially sparse regions and functional similarity ($k_{e}\gg k_{p}$) in densely populated regions, effectively overcoming the computational challenges posed by tissue heterogeneity.

Secondly, cell velocity is defined as the probability-weighted displacement vector in the pseudo-time gradient direction. For cell i, its velocity vector calculation model is:


$$\begin{array}{c} v_{i}=\frac{\text{1}}{\left|N_{i}\right|}\sum_{j\in N_{i}}\gamma_{ij}\cdot \frac{s_{j}-s_{i}}{∥s_{j}-s_{i}∥+\epsilon } \end{array}$$ (34)

where $s_{i}\;=\left(x_{i}\;,y_{i}\;\right)$ denotes the cell spatial coordinate, $\gamma_{ij}$ is the direction correction factor (when $\tau_{j}\geq \tau_{i}$, take $p_{ij}$, otherwise take $-p_{ij}$), $p_{ij}=P_{ij}/\sum_{k\in N_{i}}P_{ik}$ is the normalized transition probability, is the similarity weight between cells i and j, $\tau_{i}$ is the pseudotime value, and $\varepsilon =10^{-10}$ is the numerical stability constant. This model uses pseudo-time correction to ensure that the velocity direction aligns with developmental timing, while employing transition probabilities to quantify the strength of cell state transitions.

To visualize continuous trajectories, an adaptive Gaussian kernel is used to interpolate discrete velocities into a grid space:


$$\begin{array}{c} v_{g}=\frac{\sum_{k=\text{1}}^{K}\phi (∥g-s_{k}∥)v_{k}}{max(\text{1},\sum_{k=\text{1}}^{K}\phi (∥g-s_{k}∥))}, \end{array}$$ (35)

where g is the coordinate of the grid point, the Gaussian kernel function is $\phi (d)=exp\left(-d^{\text{2}}/\left(\text{2}\sigma^{\text{2}}\right)\right)$, and the smoothing factor σ is dynamically adjusted to:


$$\begin{array}{c} \sigma =\frac{\left(X_{max}-X_{min}\right)+\left(Y_{max}-Y_{min}\right)}{\text{2}}\times \frac{\text{1}}{\left\lfloor b\cdot Q\right\rfloor -\text{1}}\times e, \end{array}$$ (36)

where $X_{min},X_{max},Y_{min},Y_{max}$ represents the extreme value of the cell spatial coordinates, b represents the base grid resolution (default 50), Q represents the density scaling factor (default 1.0), and e represents the global smoothing coefficient (default 0.5). This adaptive mechanism maintains spatial continuity while adjusting the local resolution through the density parameter Q, enabling precise modeling of heterogeneous tissues. Finally, developmental trajectories are generated through the streamline integration of the grid velocity field.

## Supporting information

S1 Note LAP Extraction and Lineage Pseudotime Construction. (DOCX)

S2 Note Generalized additive model fitting. (DOCX)

S3 Note Data preprocessing. (DOCX)

S4 Note The two-stage training loss function comprises four primary loss terms. (DOCX)

S5 Note Parameter settings. (DOCX)

S1 Table Summary of the real-space transcriptomics RNA-seq datasets used in this study. (DOCX)

S2 Table Summary of the simulated dataset used in this study. (DOCX)

S3 Table Properties of Trajectory Inference Methods. (DOCX)

S1 Fig Comparison of trajectory inference results between StPedf and SpaTrack on the multi-section simulated dataset (Sim5). a. Visualization of StPedf on the shared UMAP embedding: from left to right, cells colored by time point, cells colored by ground-truth lineage, globally mapped pseudotime inferred by StPedf, and inferred spatial trajectories reconstructed by StPedf. b. Pseudotime coloring of StPedf in the original spatial coordinates of each time-point section (T1–T4), showing the continuous change of pseudotime along the differentiation path within each section. c. Inferred spatial trajectories of StPedf in the shared UMAP embedding for each time-point section (T1–T4). d. Visualization of SpaTrack on the shared UMAP embedding: from left to right, cells colored by time point, cells colored by ground-truth lineage, globally mapped pseudotime inferred by SpaTrack, and inferred spatial trajectories reconstructed by SpaTrack. e. Pseudotime coloring of SpaTrack in the original spatial coordinates of each time-point section (T1–T4). f. Inferred spatial trajectories of SpaTrack in the shared UMAP embedding for each time-point section (T1–T4).
(DOCX)

S2 Fig Global projected pseudotime distribution of different lineages in Sim5. (DOCX)

S3 Fig Global pseudotime accuracy on Sim5. (DOCX)

S4 Fig Stability analysis of StPedf on simulated datasets. Effects of different random seeds on pseudotime inference results by StPedf. For each of the five simulated datasets, the method was run independently 30 times with distinct random seeds from 1 to 30. Spearman and Kendall correlation coefficients were calculated between the inferred pseudotime and the ground-truth time. Boxplots show the distribution of correlation coefficients across datasets, with overlaid scatter points representing results from each independent run.
(DOCX)

S5 Fig Stability analysis of StPedf on simulated datasets. Effects of different random seeds on pseudotime inference results by StPedf. For each of the five simulated datasets, the method was run independently 30 times with distinct random seeds from 1 to 30. Spearman and Kendall correlation coefficients were calculated between the inferred pseudotime and the ground-truth time. Boxplots show the distribution of correlation coefficients across datasets, with overlaid scatter points representing results from each independent run.
(DOCX)

S6 Fig Comparison of StPedf with DPT on embedding and PAGA on embedding on Simulated Datasets. Performance was evaluated by comparing Spearman correlations and Kendall’s rank correlation coefficients across three experiments: StPedf, DPT on embedding and PAGA on embedding.
(DOCX)

S7 Fig Comparison of StPedf with DPT on embedding and PAGA on embedding on Simulated Datasets. Performance was evaluated by comparing Spearman correlations and Kendall’s rank correlation coefficients across three experiments: StPedf, DPT on embedding and PAGA on embedding.
(DOCX)

S8 Fig Content related to *Ambystoma mexicanum* regeneration trajectories. a. Presents the spatial trajectories and corresponding optimal paths of three major lineage patterns (Lineage 1, Lineage 2, Lineage 3). Different colors and line styles indicate the spatial distribution and path characteristics of each lineage. b. Heatmaps of gene expression trajectories for each lineage (Lineage 1, Lineage 2, Lineage 3), illustrating trajectory changes at the gene expression level across lineages.
(DOCX)

S9 Fig Pseudotime-dependent genes in *Ambystoma mexicanum* lineage 1 (inferred by iSORT). Expression trends of *SLC1A3, VIM, GFAP, SPARC* reveal via GAM fitting.
(DOCX)

S10 Fig Comparison of pseudo-spatiotemporal and trajectory analyses between StPedf and other methods on the *Ambystoma mexicanum* telencephalon regeneration dataset. a. Pseudo-spatiotemporal maps inferred by Monocle, Slingshot, SpaceFlow, iSORT, and StPedf. b. Inferred spatial trajectories reconstructed by iSORT and StPedf.
(DOCX)

S11 Fig Spatial Expression Patterns of Target Genes. Scatter plots showing the spatial expression of *EPCAM, CDH1* and *CD44* genes. Each plot uses distinct colors to represent gene expression levels, with the color bar on the right indicating the numerical range of expression quantities. These plots demonstrate the differences in expression distribution of the corresponding genes within the tissue space.
(DOCX)

S12 Fig Comparison of pseudo-spatiotemporal and trajectory analyses between StPedf and other methods on the ICC dataset. a. Pseudo-spatiotemporal maps inferred by Monocle, Slingshot, SpaceFlow, iSORT, and StPedf. b. Comparison of inferred spatial trajectories reconstructed by iSORT and StPedf.
(DOCX)

S13 Fig Details of tumor regeneration trajectories. a. Spatial trajectories and corresponding optimal paths for the three major lineage patterns (Lineage 1, Lineage 2, Lineage 3). b. Gene expression trajectory heatmaps for each lineage (Lineage 1, Lineage 2, Lineage 3), illustrating trajectory changes at the gene expression level across different lineages.
(DOCX)

S14 Fig Pseudotime-dependent genes of ICC lineage 2 inferred by ISORT. Expression trends of pseudotime-dependent genes (*SAA1, SAA2, MMP7, CD74, NRG1*) in lineage 2 are identified by fitting a generalized additive model (GAM).
(DOCX)

S15 Fig Enrichment analysis results for gene sets of pseudo-temporally correlated genes in the DLPFC. (DOCX)

S16 FigSensitivity analysis of hyperparameters in the model.a. Sensitivity to the diagonal term parameter. The diagonal term parameter is varied from $10^{\text{5}}$ to $10^{\text{9}}$, with $10^{\text{7}}$ set as the baseline. For each value, the corresponding optimal transport (OT) matrix is computed, and the Pearson correlation coefficient (PCC) is calculated between this matrix and the OT matrix obtained under the baseline parameter. b. Sensitivity to the regularization parameter reg. The regularization parameter reg is varied from 0.1 to 1 in steps of 0.1. For each reg value, the corresponding OT matrix is computed, and the PCC between this matrix and the OT matrix obtained with reg = 0.1 (used as the baseline) is calculated. c. Sensitivity to the alpha_range (min) parameter. The alpha_range (min) parameter is varied from 0.1 to 1, with 0.7 set as the baseline. For each value, the corresponding OT matrix is computed, and the PCC is calculated between this matrix and the OT matrix obtained under the baseline parameter.(DOCX)

S17 Fig Ablation study of the adaptive explicit spatial fusion module in real datasets. a. Inferred spatial trajectories obtained after removing the adaptive explicit spatial fusion module on the *Ambystoma mexicanum* telencephalon regeneration dataset. b. Pseudo-spatiotemporal map obtained after removing the adaptive explicit spatial fusion module on the *Ambystoma mexicanum* telencephalon regeneration dataset. c. Inferred spatial trajectories obtained after removing the adaptive explicit spatial fusion module on the ICC tumor dataset. d. Pseudo-spatiotemporal map obtained after removing the adaptive explicit spatial fusion module on the ICC tumor dataset.
(DOCX)

S18 Fig Ablation study of the clustering loss on simulated datasets. a. Comparison of Spearman correlation coefficients between the full model (StPedf) and the variant without clustering loss across four simulated datasets. b. Comparison of Kendall correlation coefficients between the full model (StPedf) and the variant without clustering loss across four simulated datasets.
(DOCX)
