# Supplementary material for: StPedf: Cell trajectory inference of spatial transcriptomics via spatial proximity embedding and spatial density-adaptive fusion
Source: PLoS Comput Biol. 2026 Jun 5;22(6):e1014346. doi: 10.1371/journal.pcbi.1014346 (PMC13240877; doi:10.1371/journal.pcbi.1014346)
Supplement: S1 Note — (DOCX) [file pcbi.1014346.s001.docx]

**S1 Note: LAP Extraction and Lineage Pseudotime Construction**

After estimating the cell transition probabilities, we further employ the LAP method to extract a representative lineage path between a given starting cell and a terminal cell. We construct an intercellular transition graph based on the transition probability matrix, which serves as a representation of the local transition topology. Given a starting cell and a terminal cell, an initial feasible path connecting them is generated on the transition graph. On this basis, the path is iteratively optimized by minimizing the path action, yielding an optimal path that better conforms to the local transition trends. After obtaining the LAP, we further map cells in the neighborhood of the path onto it to construct a lineage-specific pseudotemporal order. Specifically, cells adjacent to the path are identified through local neighborhood search and projected to their nearest positions on the path. The order of each cell is then determined by the cumulative arc length of its projected point relative to the starting point of the path. The arc length is normalized to the range 0–1 to obtain the lineage-specific pseudotime.
