# Supplementary material for: StPedf: Cell trajectory inference of spatial transcriptomics via spatial proximity embedding and spatial density-adaptive fusion
Source: PLoS Comput Biol. 2026 Jun 5;22(6):e1014346. doi: 10.1371/journal.pcbi.1014346 (PMC13240877; doi:10.1371/journal.pcbi.1014346)
Supplement: S2 Note — (DOCX) [file pcbi.1014346.s002.docx]

**S2 Note: Generalized additive model fitting**

To characterize gene expression dynamics along pseudotime, we applied a generalized additive model (GAM) to fit the relationship between gene expression and pseudotime. For each lineage, we assessed the fitting performance and statistical significance of the association between individual genes and pseudotime, and significant dynamic genes were identified based on multiple-testing-corrected results. In our analysis pipeline, significant genes were defined using the thresholds of model fit > 0.2 and false discovery rate (FDR) < 0.05.
