# Supplementary material for: StPedf: Cell trajectory inference of spatial transcriptomics via spatial proximity embedding and spatial density-adaptive fusion
Source: PLoS Comput Biol. 2026 Jun 5;22(6):e1014346. doi: 10.1371/journal.pcbi.1014346 (PMC13240877; doi:10.1371/journal.pcbi.1014346)
Supplement: S3 Note — (DOCX) [file pcbi.1014346.s003.docx]

**S3 Note: Data preprocessing**

All single-cell RNA sequencing (scRNA-seq) and spatial transcriptomics datasets in this study are subjected to unified quality control and preprocessing using Python (version 3.9) with the Scanpy package (version 1.9.1). The specific steps are as follows:

Gene filtering: To ensure the reliability of the analysis, genes expressed in fewer than 10 cells are first removed to reduce the impact of technical noise on downstream analyses.

Normalization and log transformation: To eliminate differences in sequencing depth across cells, the total expression counts of each cell are normalized to 10,000. Following normalization, the expression values are transformed using the natural logarithm to stabilize variance and make the data more closely approximate a normal distribution, facilitating subsequent modeling.

Selection of highly variable genes: To balance computational efficiency with the retention of biological information, the top 2,000 most variable genes are used for each dataset. The Seurat v3 method is employed to model the relationship between variance and mean expression based on raw counts, and the 2,000 highly variable genes are selected. For datasets with fewer than 2,000 genes in total, all genes are retained as highly variable genes.

Data scaling: To bring different genes onto a comparable scale, the expression values of each gene are Z-score normalized to have a mean of 0 and a variance of 1, thereby preventing highly expressed genes from dominating subsequent dimensionality reduction analyses.

Dimensionality reduction via principal component analysis (PCA): to extract the main directions of variation in the data and reduce dimensionality, principal component analysis is performed on the scaled expression matrix, with the first 50 principal components retained as feature inputs for the subsequent StPedf model.

The above preprocessing pipeline ensures that datasets from different sources are comparable under a unified processing framework while maximizing the retention of key biological variation.
