# Supplementary material for: StPedf: Cell trajectory inference of spatial transcriptomics via spatial proximity embedding and spatial density-adaptive fusion
Source: PLoS Comput Biol. 2026 Jun 5;22(6):e1014346. doi: 10.1371/journal.pcbi.1014346 (PMC13240877; doi:10.1371/journal.pcbi.1014346)
Supplement: S4 Note — (DOCX) [file pcbi.1014346.s004.docx]

**S4 Note: The two-stage training loss function comprises four primary loss terms**

The first is the masked autoencoder reconstruction loss, which encourages the model to learn contextual and co-expression patterns among genes rather than simply memorizing inputs. The model reconstructs the original expression profile from partially masked inputs, with the loss being the scaled cosine error of masked nodes:

$$L_{self} =\frac{1}{\left|M\right|}\sum_{i\in M} [1-cos(\tilde{X}_{i} ,X_{i })]^{\alpha} ,\alpha=3,$$

where $M$ denotes the set of masked nodes and $\tilde{X}_{i}$ denotes the reconstructed expression vector.

The second is the global reconstruction loss, which encourages the accurate preservation of genetic information across all nodes. We simultaneously compute the mean squared error reconstruction loss for all nodes:

$$L_{rec} =\frac{1}{n}\sum_{i=1}^{n} \parallel\tilde{X}_{i} -X_{i} \parallel_{2}^{2} .$$

The third is the graph convolutional autoencoder loss, comprising a graph reconstruction loss and a variational regularization term:

where, $A$denotes the spatial adjacency matrix, $z_{i}=\mu_{i}+\varepsilon\odot\sigma_{i}$ represents the sampled graph embedding, and $\frac{\left|E\right| }{n^{2}}$ is the normalization coefficient. This loss function ensures that the embedding simultaneously aligns with the spatial graph structure and exhibits a smooth latent distribution.

The fourth is the deep clustering loss, which is based on deep embedding clustering algorithms. By minimizing the Kullback-Leibler divergence between the soft assignment $Q$and the target distribution $P$, it guides the embedding space to form a compact clustering structure:

where $C$ denotes the preset number of clusters, and $\mu_{c}$ represents the cluster center. The target distribution $P$ provides clearer supervisory signals for cluster optimization by reinforcing high-confidence assignments.
