# Supplementary material for: StPedf: Cell trajectory inference of spatial transcriptomics via spatial proximity embedding and spatial density-adaptive fusion
Source: PLoS Comput Biol. 2026 Jun 5;22(6):e1014346. doi: 10.1371/journal.pcbi.1014346 (PMC13240877; doi:10.1371/journal.pcbi.1014346)
Supplement: S5 Note — (DOCX) [file pcbi.1014346.s005.docx]

**S5 Note: Parameter settings**

This study involves several modules, each with a set of key parameters. Their settings and rationales are summarized as follows.

Data preprocessing: Genes expressed in fewer than 10 cells are removed by setting min_cells = 10. Library size normalization is performed with target_sum = 1e4. Highly variable genes are selected using the Seurat v3 method with n_top_genes = 2000. For dimensionality reduction, principal component analysis (PCA) is applied, and the first n_comps = 50 principal components are retained.

Spatial graph construction: The spatial K-nearest neighbor (KNN) graph is constructed with n_neighbors = 12 based on spatial coordinates. The graph construction mode is set to mode = 'AlphaComplex', and the alpha-shape-related parameter is set to alpha_n_neighbors = 30.

Embedding training: The embedding model is trained in clustering mode (mode = 'clustering'), which enables the deep embedding clustering loss. The number of iterations is set to N = 500. To ensure reproducibility, the random seed is fixed at random_seed = 2025.

Adaptive transition matrix construction: The adaptive transition matrix is constructed using several key hyperparameters, selected based on dataset characteristics and optimization objectives. The Sinkhorn regularization coefficient reg is chosen via sensitivity analysis to balance the matrix's smoothness and sparsity. The diagonal penalty diag_val = 1e6 is set to prevent self-transport. The dynamic spatial weight range alpha_range = (α_min, α_max) maps normalized local density to adaptive spatial weights. Specifically, the weights approach α_min in high-density regions and α_max in low-density regions, with high-density areas emphasizing transcriptional similarity and low-density areas emphasizing spatial continuity. In practice, α_max is typically set close to 1 to preserve spatial constraints in sparse regions, while α_min is chosen according to the degree of spatial density heterogeneity in each dataset. For datasets with strong density contrast, a wider range is used; for more uniform densities, a narrower range is applied to stabilize the transition matrix. Kernel density estimation is performed with a fixed bandwidth of 50. The spot_size = 50 parameter is used for visualization only and does not affect model construction.

Trajectory inference: The starting cells are selected using select_way = 'cell_type', in which specified clusters are used as the initial cell groups. The number of spatial neighbors is set to n_neigh = 30. For velocity field estimation, the number of neighbors is set to n_neigh_pos = 50. The vector field smoothing parameters are set to n_neighbors = 80 and smooth = 1.2.

Optimizer and model training: Model optimization is performed using the Adam optimizer with default settings, a learning rate of 0.001, and weight_decay = 0. The number of training epochs is adjusted according to sample size, with a default value of 600. The clustering loss weight in the overall objective function is set to 20.

Unless otherwise specified, the above parameter settings are used throughout the experiments. In addition, the robustness of major hyperparameters is further evaluated through sensitivity analysis on real datasets.
