# Supplementary material for: StPedf: Cell trajectory inference of spatial transcriptomics via spatial proximity embedding and spatial density-adaptive fusion
Source: PLoS Comput Biol. 2026 Jun 5;22(6):e1014346. doi: 10.1371/journal.pcbi.1014346 (PMC13240877; doi:10.1371/journal.pcbi.1014346)
Supplement: S1 Table — (DOCX) [file pcbi.1014346.s006.docx]

**S1 Table: Summary of the real-space transcriptomics RNA-seq datasets used in this study**

| Dataset | Tissue | Organism | Time points | Samples | Platforms | Source |
| --- | --- | --- | --- | --- | --- | --- |
| Axolotl dataset | Axolotl brain | *Ambystoma mexicanum* | 5, 10, 15, 20 days after injury | 4 time - point samples | Stereo-seq | CNGB Nucleotide Sequence Archive (Accession number: [CNP0002068](https://db.cngb.org/data_resources/project/CNP0002068)) |
| Tumor dataset | Liver bile duct | Human | - | 1 | Stereo-seq | CNGBdb Database (Accession number: [CNP0002199](https://db.cngb.org/data_resources/project/CNP0002199)) |
| DLPFC (151673) | Dorsolateral prefrontal cortex | Human | - | 1 | 10x Visium | <http://research.libd.org/spatialLIBD/> |
