# Supplementary material for: StPedf: Cell trajectory inference of spatial transcriptomics via spatial proximity embedding and spatial density-adaptive fusion
Source: PLoS Comput Biol. 2026 Jun 5;22(6):e1014346. doi: 10.1371/journal.pcbi.1014346 (PMC13240877; doi:10.1371/journal.pcbi.1014346)
Supplement: S2 Table — (DOCX) [file pcbi.1014346.s007.docx]

**S2 Table: Summary of the simulated dataset used in this study**

| Simulated Dataset | Cells | Genes | Trajectory Type | Spatial Pattern | Source | Cell Types | Cell Type Composition | Source Tool |
| --- | --- | --- | --- | --- | --- | --- | --- | --- |
| Simulated Dataset 1 | 1000 | 135 | Bifurcated Structure | - | Generated based on dyngen (default bifurcated backbone) | 10 | Cell Types A - G (Lineage 1), H - J (Lineage 2) | dyngen |
| Simulated Dataset 2 | 500 | 100 | Trifurcated Structure (Linear Spatial Pattern) | Linear | Generated based on dyngen (binary tree backbone) | 10 | Cell Types A - F (Lineage 1), G - H (Lineage 2), I - J (Lineage 3) | dyngen |
| Simulated Dataset 3 | 500 | 100 | Trifurcated Structure (Non - Linear Spatial Pattern) | Non - linear | Generated based on dyngen (binary tree backbone) | 10 | Cell Types A - F (Lineage 1), G - H (Lineage 2), I - J (Lineage 3) | dyngen |
| Simulated Dataset 4 | 454 | 100 | Independent Double - Path Structure | - | Generated based on dyngen (broken backbone) | 6 | Cells from two unrelated lineages | dyngen |
| Simulated Dataset 5 | 4800 | 2000 | Progressive bifurcating structure across four time points | Multi-slice spatial pattern with slice-specific geometric transformations | Custom simulated dataset generated with four overlapping time windows, branching lineages, and slice-specific spatial transformations | 3 | Prog, Path1, and Path2 across T1–T4 | Python |
