# Supplementary material for: StPedf: Cell trajectory inference of spatial transcriptomics via spatial proximity embedding and spatial density-adaptive fusion
Source: PLoS Comput Biol. 2026 Jun 5;22(6):e1014346. doi: 10.1371/journal.pcbi.1014346 (PMC13240877; doi:10.1371/journal.pcbi.1014346)
Supplement: S3 Table — (DOCX) [file pcbi.1014346.s008.docx]

**S3 Table: Properties of Trajectory Inference Methods**

| Method | Last Update | Pseudotime | Spatial Localization | Scalability | Applicable Data Types | Complex Topology | Software Language |
| --- | --- | --- | --- | --- | --- | --- | --- |
| DPT | 2016 | Yes | No | High | Single - cell transcriptome data | Yes | Python |
| Moncle 3 | 2019 | Yes | No | Medium | Single - cell transcriptome data, spatial transcriptome data | Yes | R |
| TSCAN | 2016 | Yes | No | Medium | Single - cell transcriptome data | Yes | R |
| SpaTrack | 2025 | Yes | No | High | Spatial transcriptome data | Yes | Python |
| Spaceflow | 2022 | Yes | No | High | Spatial transcriptome data | Yes | Python |
| SpatialPCA | 2022 | Yes | No | High | Spatial transcriptome data | Yes | R |
