# Supplementary material for: StPedf: Cell trajectory inference of spatial transcriptomics via spatial proximity embedding and spatial density-adaptive fusion
Source: PLoS Comput Biol. 2026 Jun 5;22(6):e1014346. doi: 10.1371/journal.pcbi.1014346 (PMC13240877; doi:10.1371/journal.pcbi.1014346)
Supplement: S1 Fig — a. Visualization of StPedf on the shared UMAP embedding: from left to right, cells colored by time point, cells colored by ground-truth lineage, globally mapped pseudotime inferred by StPedf, and inferred spatial trajectories reconstructed by StPedf. b. Pseudotime coloring of StPedf in the original spatial coordinates of each time-point section (T1–T4), showing the continuous change of pseudotime along the differentiation path within each section. c. Inferred spatial trajectories of StPedf in the shared UMAP embedding for each time-point section (T1–T4). d. Visualization of SpaTrack on the shared UMAP embedding: from left to right, cells colored by time point, cells colored by ground-truth lineage, globally mapped pseudotime inferred by SpaTrack, and inferred spatial trajectories reconstructed by SpaTrack. e. Pseudotime coloring of SpaTrack in the original spatial coordinates of each time-point section (T1–T4). f. Inferred spatial trajectories of SpaTrack in the shared UMAP embedding for each time-point section (T1–T4). (DOCX) [file pcbi.1014346.s009.docx]

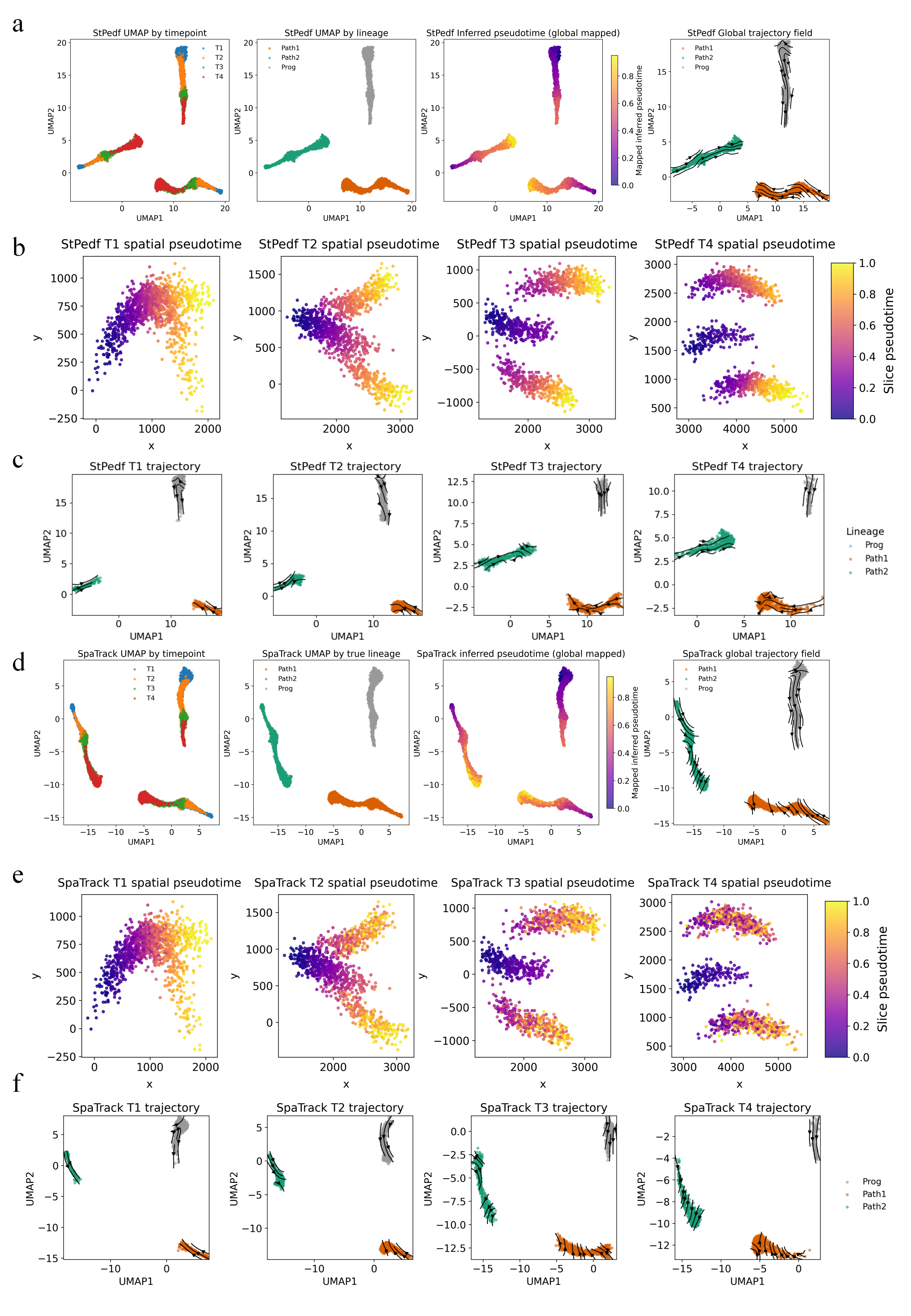


**Fig S1. Comparison of trajectory inference results between StPedf and SpaTrack on the multi-section simulated dataset (Sim5).**

**a.** Visualization of StPedf on the shared UMAP embedding: from left to right, cells colored by time point, cells colored by ground-truth lineage, globally mapped pseudotime inferred by StPedf, and inferred spatial trajectories reconstructed by StPedf.
**b.** Pseudotime coloring of StPedf in the original spatial coordinates of each time-point section (T1–T4), showing the continuous change of pseudotime along the differentiation path within each section.
**c.** Inferred spatial trajectories of StPedf in the shared UMAP embedding for each time-point section (T1–T4).
**d.** Visualization of SpaTrack on the shared UMAP embedding: from left to right, cells colored by time point, cells colored by ground-truth lineage, globally mapped pseudotime inferred by SpaTrack, and inferred spatial trajectories reconstructed by SpaTrack.
**e.** Pseudotime coloring of SpaTrack in the original spatial coordinates of each time-point section (T1–T4).
**f.** Inferred spatial trajectories of SpaTrack in the shared UMAP embedding for each time-point section (T1–T4).
