# Supplementary figures and images for: StPedf: Cell trajectory inference of spatial transcriptomics via spatial proximity embedding and spatial density-adaptive fusion
Source: PLoS Comput Biol. 2026 Jun 5;22(6):e1014346. doi: 10.1371/journal.pcbi.1014346 (PMC13240877; doi:10.1371/journal.pcbi.1014346)

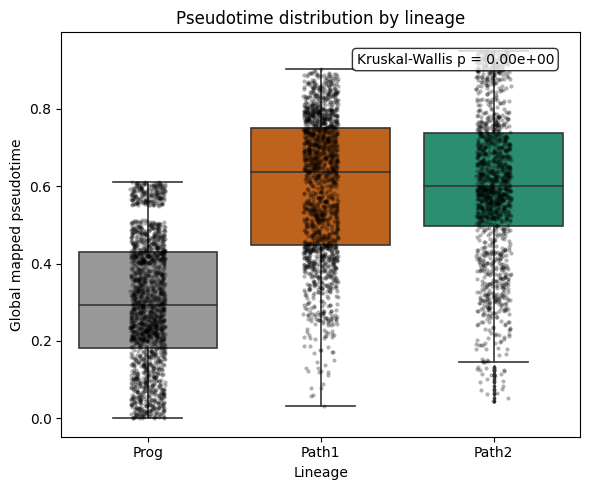


**S2 Fig. Global projected pseudotime distribution of different lineages in Sim5.**

Supplement: S2 Fig — (DOCX) [file pcbi.1014346.s010.docx]

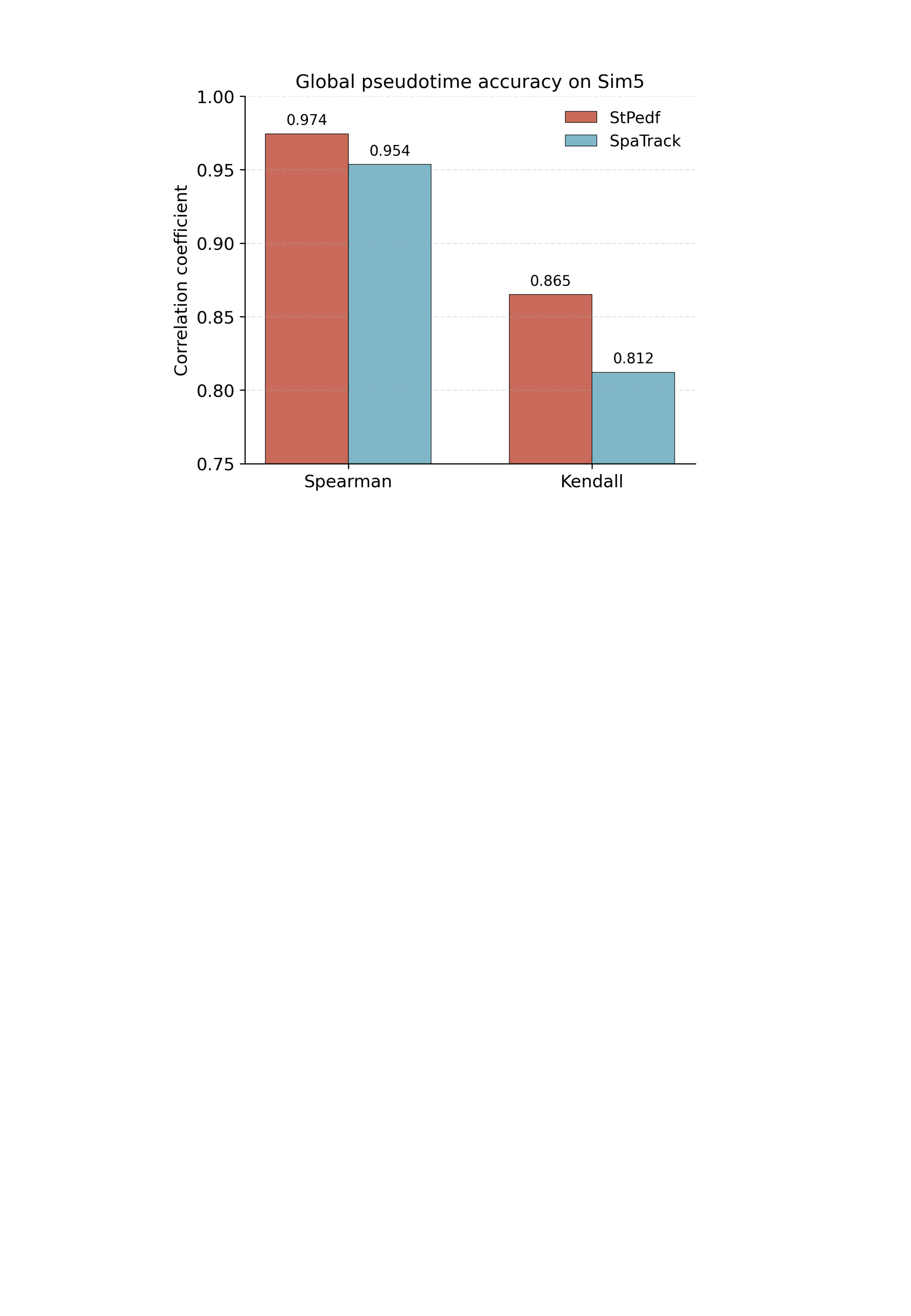


**S3 Fig. Global pseudotime accuracy on Sim5.**

Supplement: S3 Fig — (DOCX) [file pcbi.1014346.s011.docx]
