# Supplementary material for: StPedf: Cell trajectory inference of spatial transcriptomics via spatial proximity embedding and spatial density-adaptive fusion
Source: PLoS Comput Biol. 2026 Jun 5;22(6):e1014346. doi: 10.1371/journal.pcbi.1014346 (PMC13240877; doi:10.1371/journal.pcbi.1014346)
Supplement: S4 Fig — Effects of different random seeds on pseudotime inference results by StPedf. For each of the five simulated datasets, the method was run independently 30 times with distinct random seeds from 1 to 30. Spearman and Kendall correlation coefficients were calculated between the inferred pseudotime and the ground-truth time. Boxplots show the distribution of correlation coefficients across datasets, with overlaid scatter points representing results from each independent run. (DOCX) [file pcbi.1014346.s012.docx]

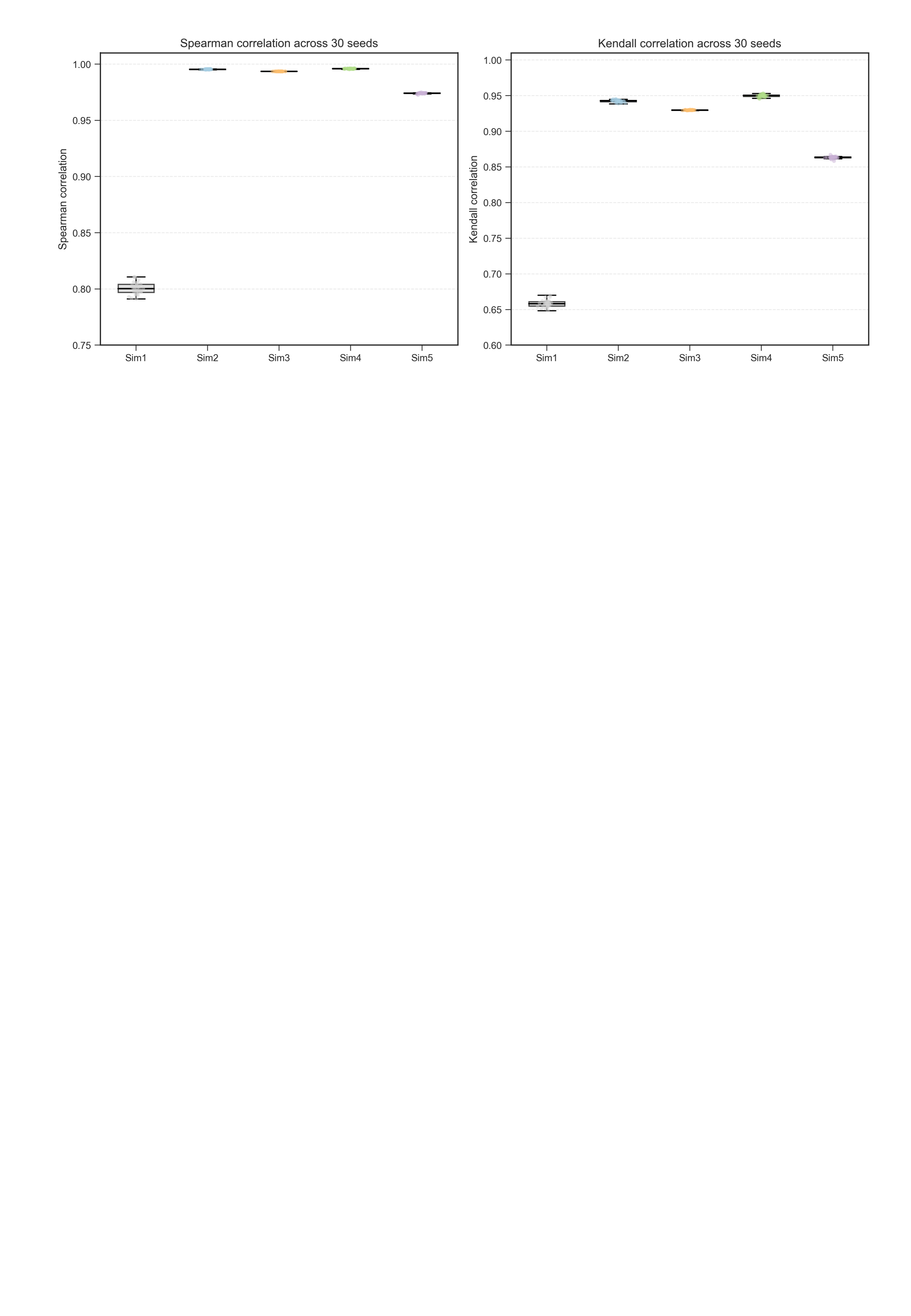


**S4 Fig. Stability analysis of StPedf on simulated datasets.** Effects of different random seeds on pseudotime inference results by StPedf. For each of the five simulated datasets, the method was run independently 30 times with distinct random seeds from 1 to 30. Spearman and Kendall correlation coefficients were calculated between the inferred pseudotime and the ground-truth time. Boxplots show the distribution of correlation coefficients across datasets, with overlaid scatter points representing results from each independent run.
