# Supplementary material for: StPedf: Cell trajectory inference of spatial transcriptomics via spatial proximity embedding and spatial density-adaptive fusion
Source: PLoS Comput Biol. 2026 Jun 5;22(6):e1014346. doi: 10.1371/journal.pcbi.1014346 (PMC13240877; doi:10.1371/journal.pcbi.1014346)
Supplement: S6 Fig — Performance was evaluated by comparing Spearman correlations and Kendall’s rank correlation coefficients across three experiments: StPedf, DPT on embedding and PAGA on embedding. (DOCX) [file pcbi.1014346.s014.docx]

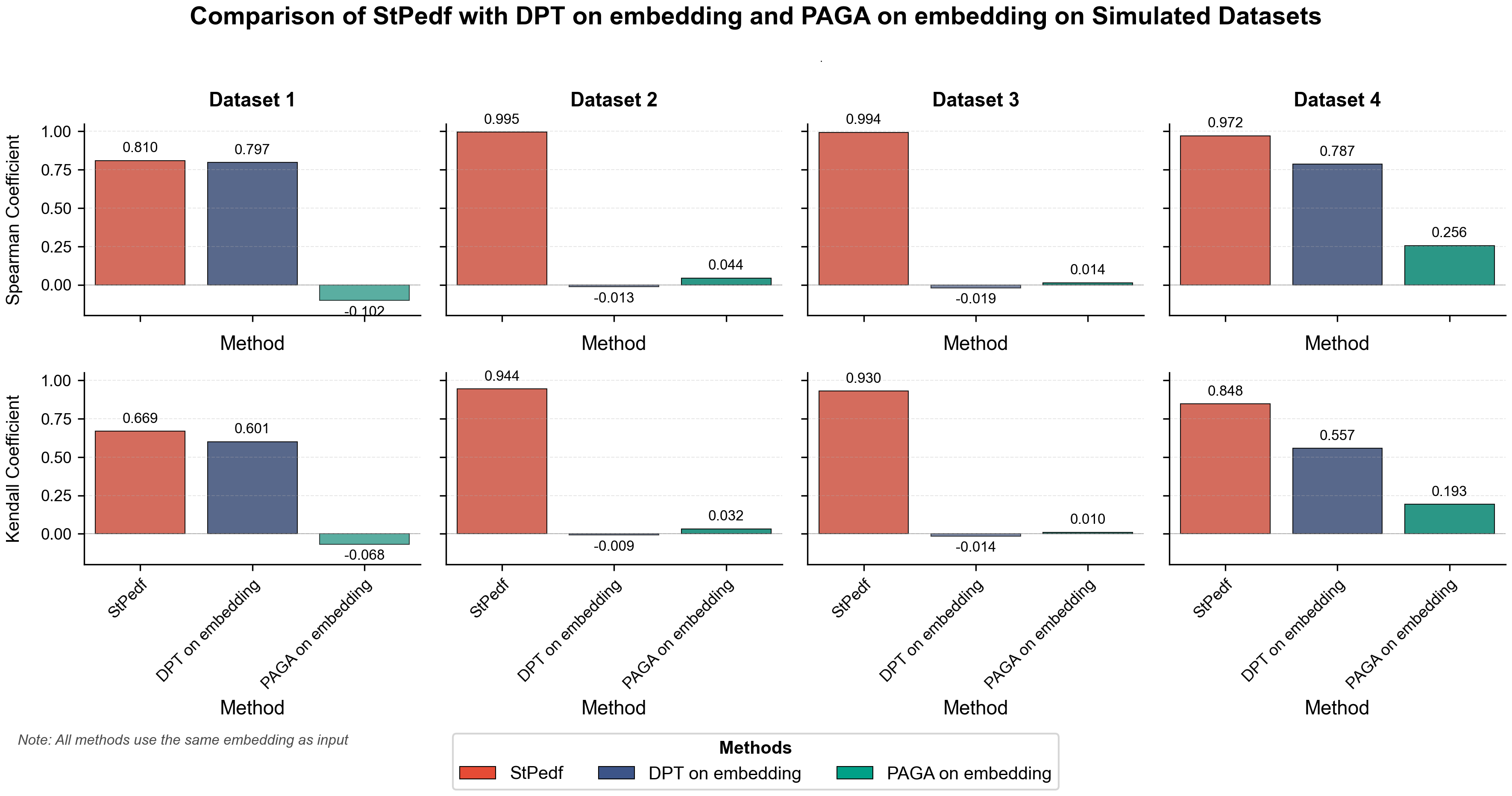


**S6 Fig. Comparison of StPedf with DPT on embedding and PAGA on embedding on Simulated Datasets.** Performance was evaluated by comparing Spearman correlations and Kendall's rank correlation coefficients across three experiments: StPedf, DPT on embedding and PAGA on embedding.
