# Supplementary material for: StPedf: Cell trajectory inference of spatial transcriptomics via spatial proximity embedding and spatial density-adaptive fusion
Source: PLoS Comput Biol. 2026 Jun 5;22(6):e1014346. doi: 10.1371/journal.pcbi.1014346 (PMC13240877; doi:10.1371/journal.pcbi.1014346)
Supplement: S7 Fig — Performance was evaluated by comparing Spearman correlations and Kendall’s rank correlation coefficients across three experiments: StPedf, DPT on embedding and PAGA on embedding. (DOCX) [file pcbi.1014346.s015.docx]

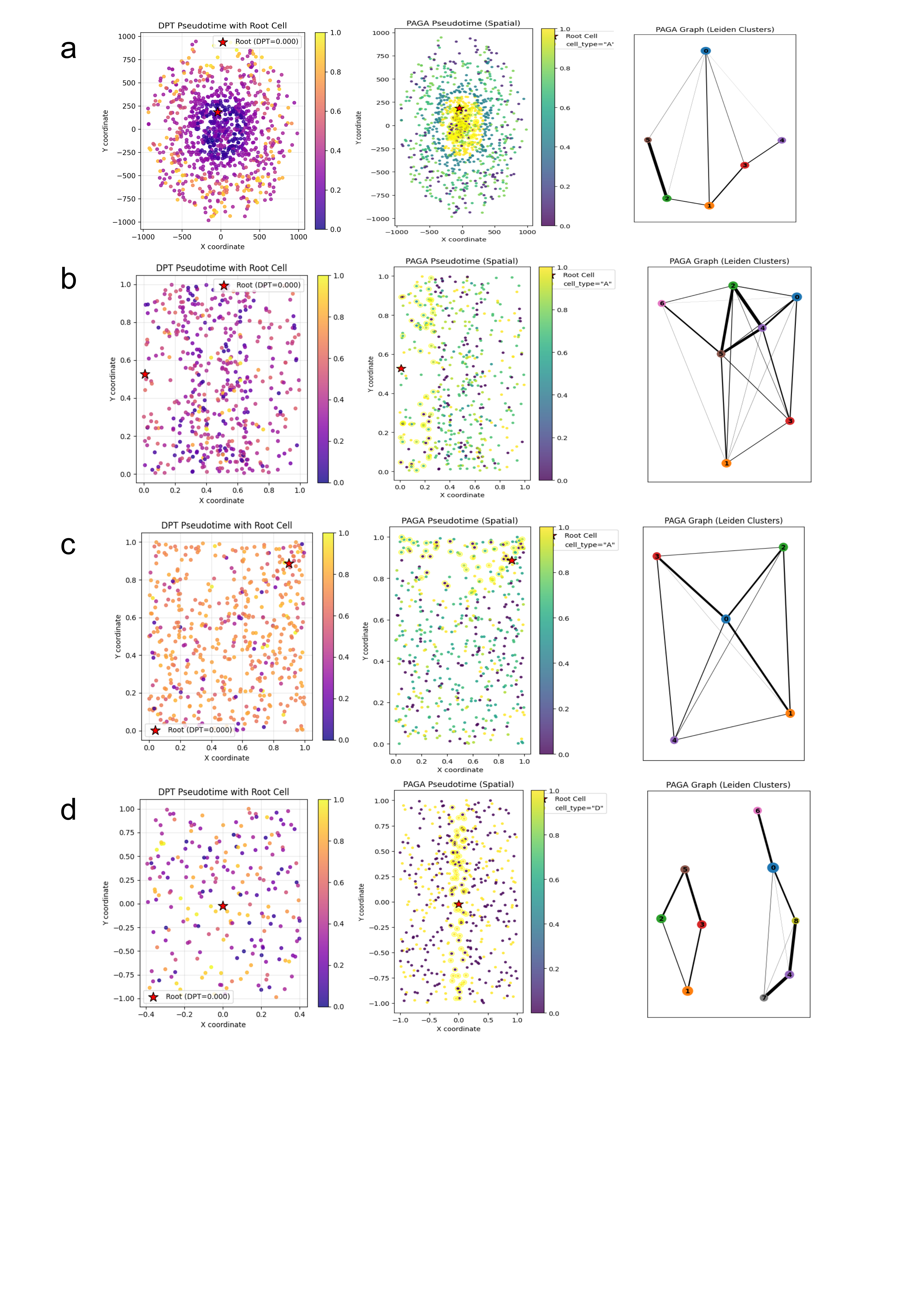


**S7 Fig.** **Qualitative comparison of pseudotime inference results across four simulated datasets.** a-d. Each panel corresponds to one simulated dataset, presenting three types of results for that dataset: Left subplot: Pseudotime distribution inferred by "same embedding + DPT" — the root cell is marked with a red star, and pseudotime values are reflected by the color gradient. Middle subplot: Spatial pseudotime distribution inferred by "same embedding + PAGA" — pseudotime values are reflected by the color gradient. Right subplot: PAGA graph of the dataset — nodes represent clusters partitioned by the Leiden algorithm, and edges indicate the connection relationships between clusters.
