# Supplementary material for: StPedf: Cell trajectory inference of spatial transcriptomics via spatial proximity embedding and spatial density-adaptive fusion
Source: PLoS Comput Biol. 2026 Jun 5;22(6):e1014346. doi: 10.1371/journal.pcbi.1014346 (PMC13240877; doi:10.1371/journal.pcbi.1014346)
Supplement: S8 Fig — a. Presents the spatial trajectories and corresponding optimal paths of three major lineage patterns (Lineage 1, Lineage 2, Lineage 3). Different colors and line styles indicate the spatial distribution and path characteristics of each lineage. b. Heatmaps of gene expression trajectories for each lineage (Lineage 1, Lineage 2, Lineage 3), illustrating trajectory changes at the gene expression level across lineages. (DOCX) [file pcbi.1014346.s016.docx]

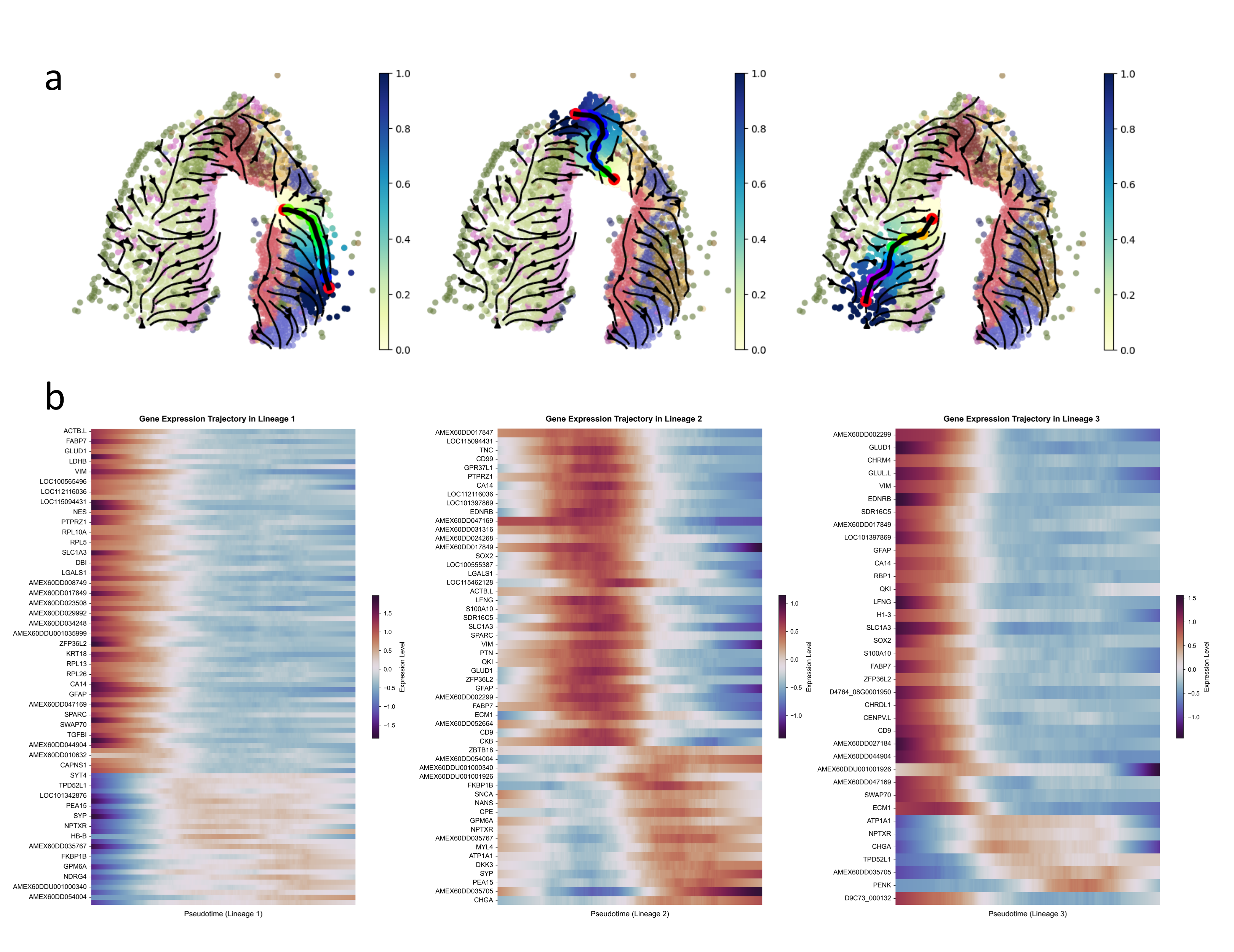


**S8 Fig. Content related to *Ambystoma mexicanum* regeneration trajectories. a.** Presents the spatial trajectories and corresponding optimal paths of three major lineage patterns (Lineage 1, Lineage 2, Lineage 3). Different colors and line styles indicate the spatial distribution and path characteristics of each lineage. **b.** Heatmaps of gene expression trajectories for each lineage (Lineage 1, Lineage 2, Lineage 3), illustrating trajectory changes at the gene expression level across lineages.
