# Supplementary material for: StPedf: Cell trajectory inference of spatial transcriptomics via spatial proximity embedding and spatial density-adaptive fusion
Source: PLoS Comput Biol. 2026 Jun 5;22(6):e1014346. doi: 10.1371/journal.pcbi.1014346 (PMC13240877; doi:10.1371/journal.pcbi.1014346)
Supplement: S9 Fig — Expression trends of SLC1A3, VIM, GFAP, SPARC reveal via GAM fitting. (DOCX) [file pcbi.1014346.s017.docx]

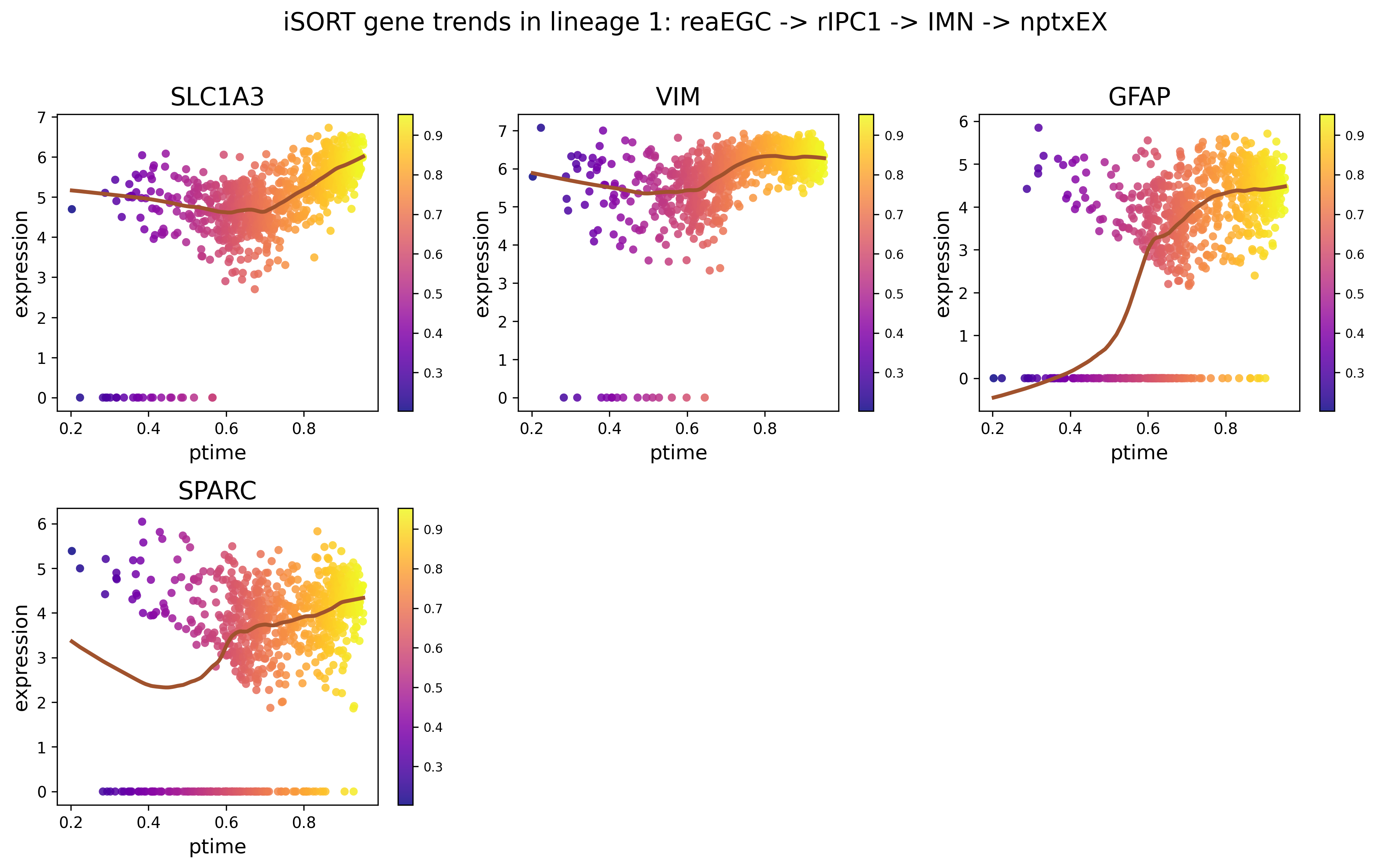


**S9 Fig. Pseudotime-dependent genes in *Ambystoma mexicanum* lineage 1 (inferred by iSORT).** Expression trends of *SLC1A3, VIM, GFAP, SPARC* reveal via GAM fitting.
