# Supplementary material for: StPedf: Cell trajectory inference of spatial transcriptomics via spatial proximity embedding and spatial density-adaptive fusion
Source: PLoS Comput Biol. 2026 Jun 5;22(6):e1014346. doi: 10.1371/journal.pcbi.1014346 (PMC13240877; doi:10.1371/journal.pcbi.1014346)
Supplement: S11 Fig — Scatter plots showing the spatial expression of EPCAM, CDH1 and CD44 genes. Each plot uses distinct colors to represent gene expression levels, with the color bar on the right indicating the numerical range of expression quantities. These plots demonstrate the differences in expression distribution of the corresponding genes within the tissue space. (DOCX) [file pcbi.1014346.s019.docx]

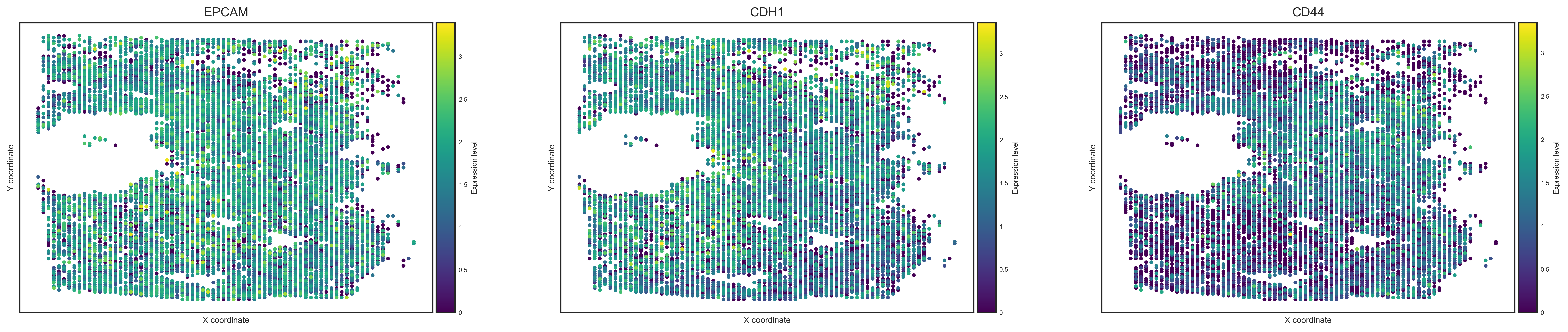


**S11 Fig. Spatial Expression Patterns of Target Genes.** Scatter plots showing the spatial expression of *EPCAM, CDH1* and *CD44* genes. Each plot uses distinct colors to represent gene expression levels, with the color bar on the right indicating the numerical range of expression quantities. These plots demonstrate the differences in expression distribution of the corresponding genes within the tissue space.
