# Supplementary material for: StPedf: Cell trajectory inference of spatial transcriptomics via spatial proximity embedding and spatial density-adaptive fusion
Source: PLoS Comput Biol. 2026 Jun 5;22(6):e1014346. doi: 10.1371/journal.pcbi.1014346 (PMC13240877; doi:10.1371/journal.pcbi.1014346)
Supplement: S12 Fig — a. Pseudo-spatiotemporal maps inferred by Monocle, Slingshot, SpaceFlow, iSORT, and StPedf. b. Comparison of inferred spatial trajectories reconstructed by iSORT and StPedf. (DOCX) [file pcbi.1014346.s020.docx]

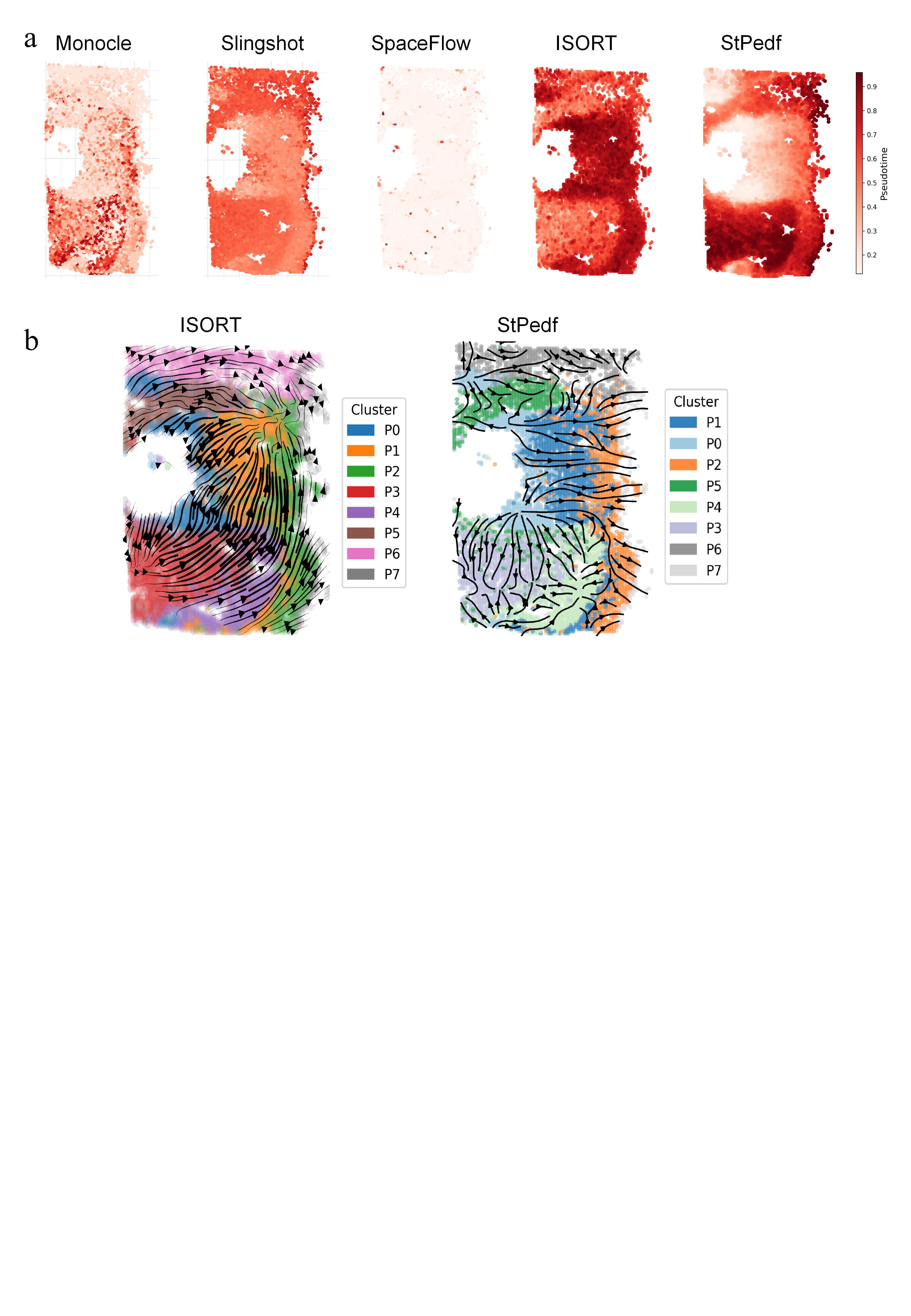


**S12 Fig. Comparison of pseudo-spatiotemporal and trajectory analyses between StPedf and other methods on the ICC dataset.**
**a.** Pseudo-spatiotemporal maps inferred by Monocle, Slingshot, SpaceFlow, iSORT, and StPedf.
**b.** Comparison of inferred spatial trajectories reconstructed by iSORT and StPedf.
