# Supplementary material for: StPedf: Cell trajectory inference of spatial transcriptomics via spatial proximity embedding and spatial density-adaptive fusion
Source: PLoS Comput Biol. 2026 Jun 5;22(6):e1014346. doi: 10.1371/journal.pcbi.1014346 (PMC13240877; doi:10.1371/journal.pcbi.1014346)
Supplement: S13 Fig — a. Spatial trajectories and corresponding optimal paths for the three major lineage patterns (Lineage 1, Lineage 2, Lineage 3). b. Gene expression trajectory heatmaps for each lineage (Lineage 1, Lineage 2, Lineage 3), illustrating trajectory changes at the gene expression level across different lineages. (DOCX) [file pcbi.1014346.s021.docx]

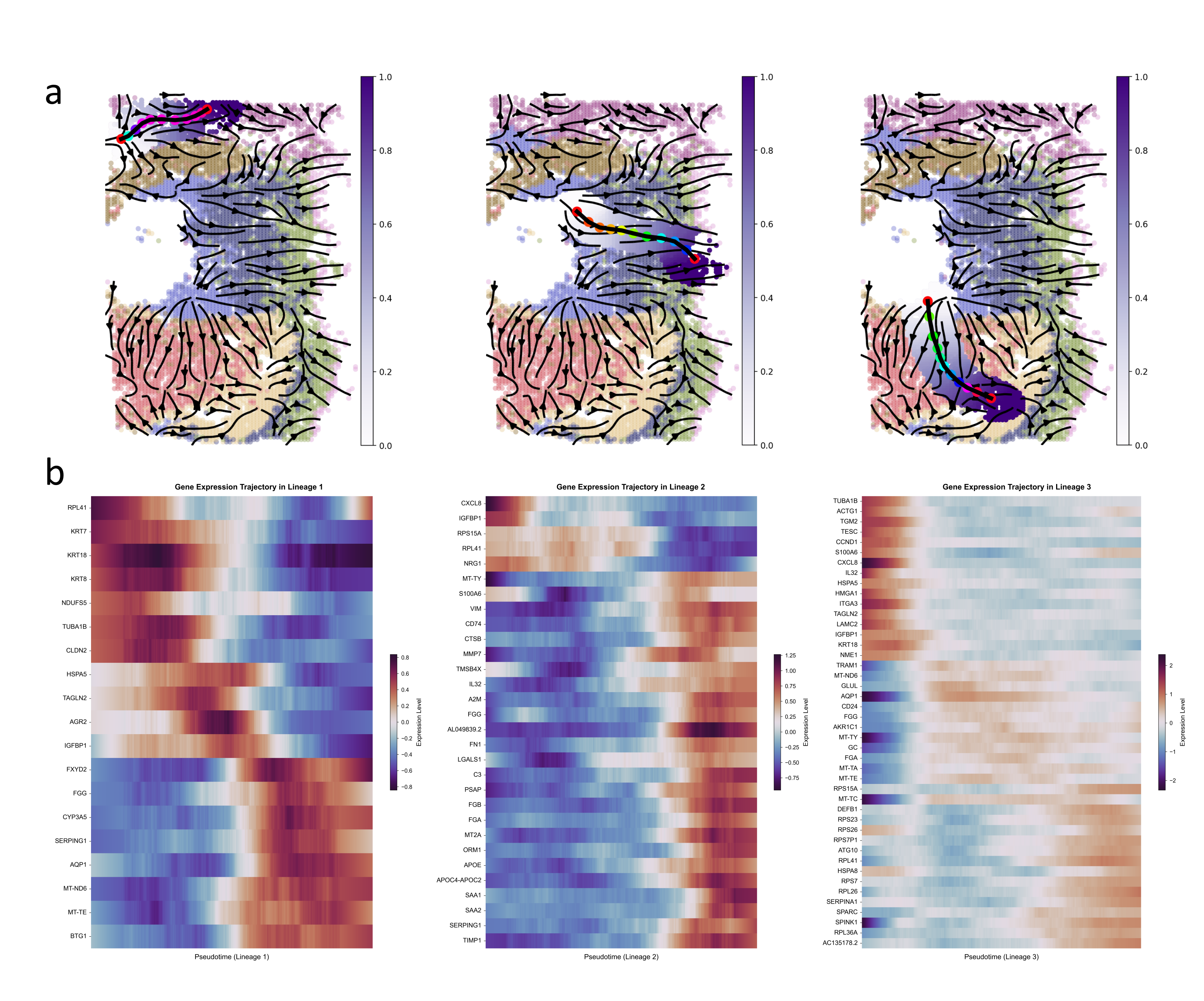


**S13 Fig. Details of tumor regeneration trajectories.** **a.** Spatial trajectories and corresponding optimal paths for the three major lineage patterns (Lineage 1, Lineage 2, Lineage 3). **b.** Gene expression trajectory heatmaps for each lineage (Lineage 1, Lineage 2, Lineage 3), illustrating trajectory changes at the gene expression level across different lineages.
