# Supplementary material for: StPedf: Cell trajectory inference of spatial transcriptomics via spatial proximity embedding and spatial density-adaptive fusion
Source: PLoS Comput Biol. 2026 Jun 5;22(6):e1014346. doi: 10.1371/journal.pcbi.1014346 (PMC13240877; doi:10.1371/journal.pcbi.1014346)
Supplement: S14 Fig — Expression trends of pseudotime-dependent genes (SAA1, SAA2, MMP7, CD74, NRG1) in lineage 2 are identified by fitting a generalized additive model (GAM). (DOCX) [file pcbi.1014346.s022.docx]

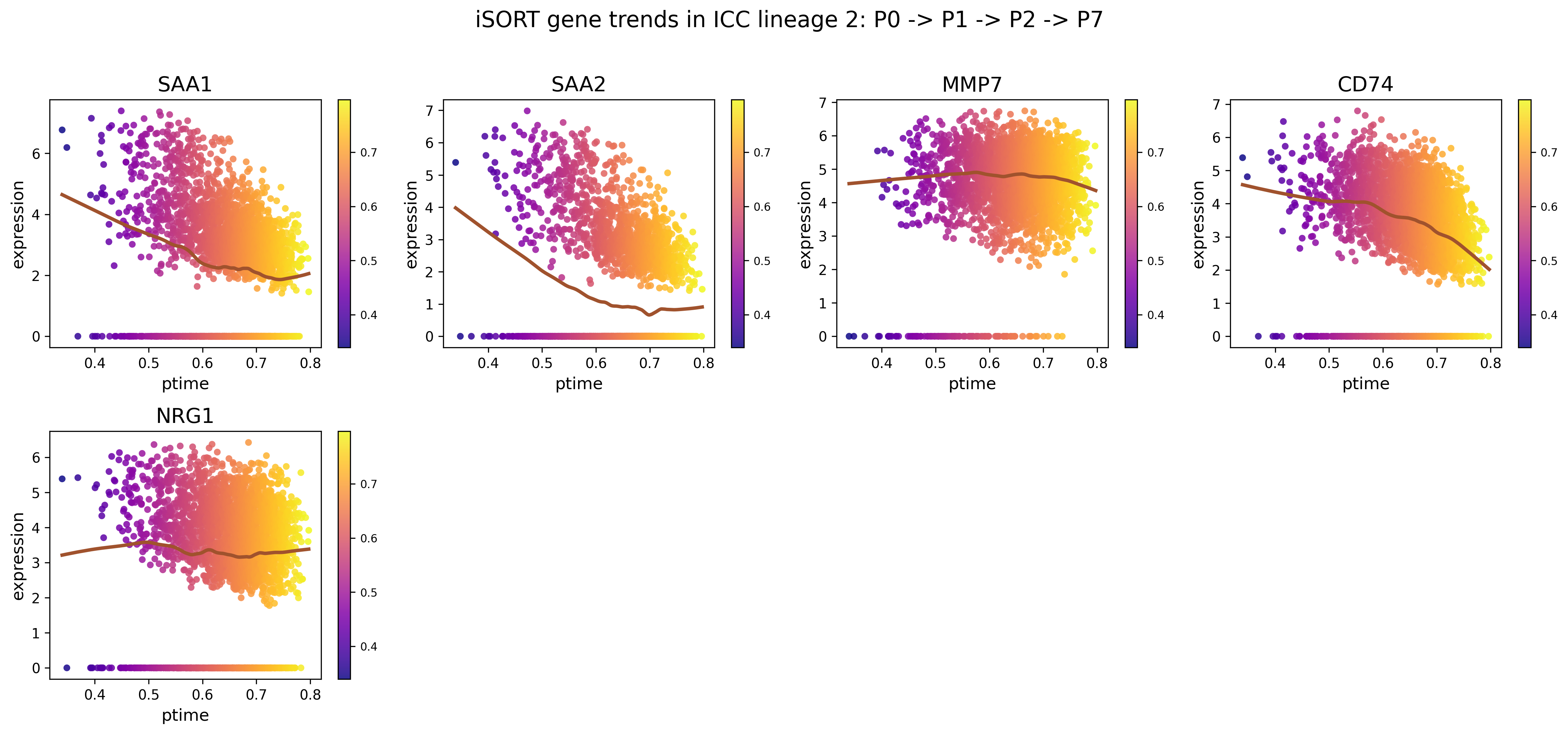


**S14 Fig. Pseudotime-dependent genes of ICC lineage 2 inferred by ISORT.**Expression trends of pseudotime-dependent genes (*SAA1, SAA2, MMP7, CD74, NRG1*) in lineage 2 are identified by fitting a generalized additive model (GAM).
