# Supplementary material for: StPedf: Cell trajectory inference of spatial transcriptomics via spatial proximity embedding and spatial density-adaptive fusion
Source: PLoS Comput Biol. 2026 Jun 5;22(6):e1014346. doi: 10.1371/journal.pcbi.1014346 (PMC13240877; doi:10.1371/journal.pcbi.1014346)
Supplement: S15 Fig — (DOCX) [file pcbi.1014346.s023.docx]

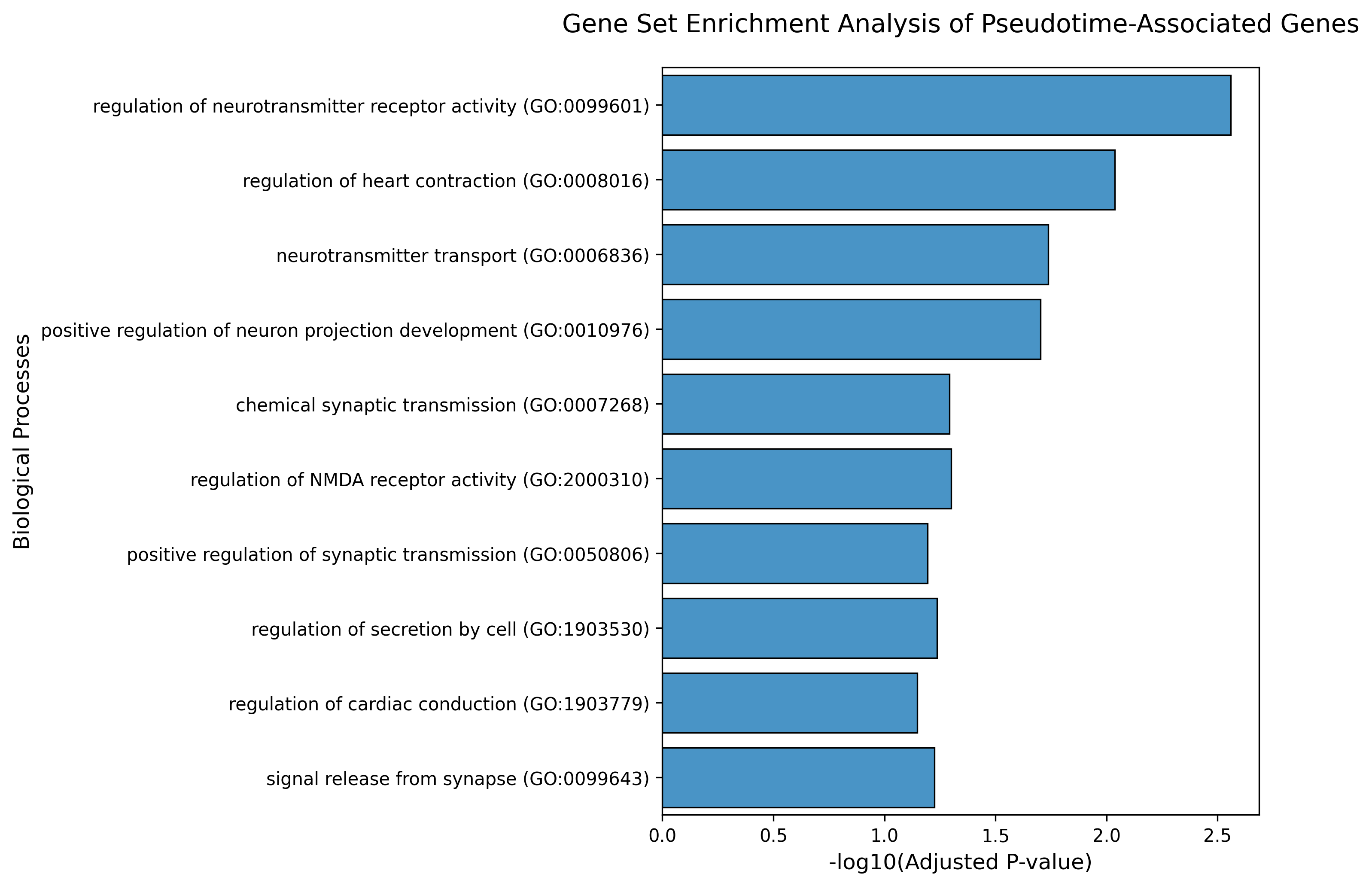


**S15 Fig.** Enrichment analysis results for gene sets of pseudo-temporally correlated genes in the DLPFC.
