# Supplementary material for: StPedf: Cell trajectory inference of spatial transcriptomics via spatial proximity embedding and spatial density-adaptive fusion
Source: PLoS Comput Biol. 2026 Jun 5;22(6):e1014346. doi: 10.1371/journal.pcbi.1014346 (PMC13240877; doi:10.1371/journal.pcbi.1014346)
Supplement: S16 Fig — a. Sensitivity to the diagonal term parameter. The diagonal term parameter is varied from 105 to 109, with 107 set as the baseline. For each value, the corresponding optimal transport (OT) matrix is computed, and the Pearson correlation coefficient (PCC) is calculated between this matrix and the OT matrix obtained under the baseline parameter. b. Sensitivity to the regularization parameter reg. The regularization parameter reg is varied from 0.1 to 1 in steps of 0.1. For each reg value, the corresponding OT matrix is computed, and the PCC between this matrix and the OT matrix obtained with reg = 0.1 (used as the baseline) is calculated. c. Sensitivity to the alpha_range (min) parameter. The alpha_range (min) parameter is varied from 0.1 to 1, with 0.7 set as the baseline. For each value, the corresponding OT matrix is computed, and the PCC is calculated between this matrix and the OT matrix obtained under the baseline parameter. (DOCX) [file pcbi.1014346.s024.docx]

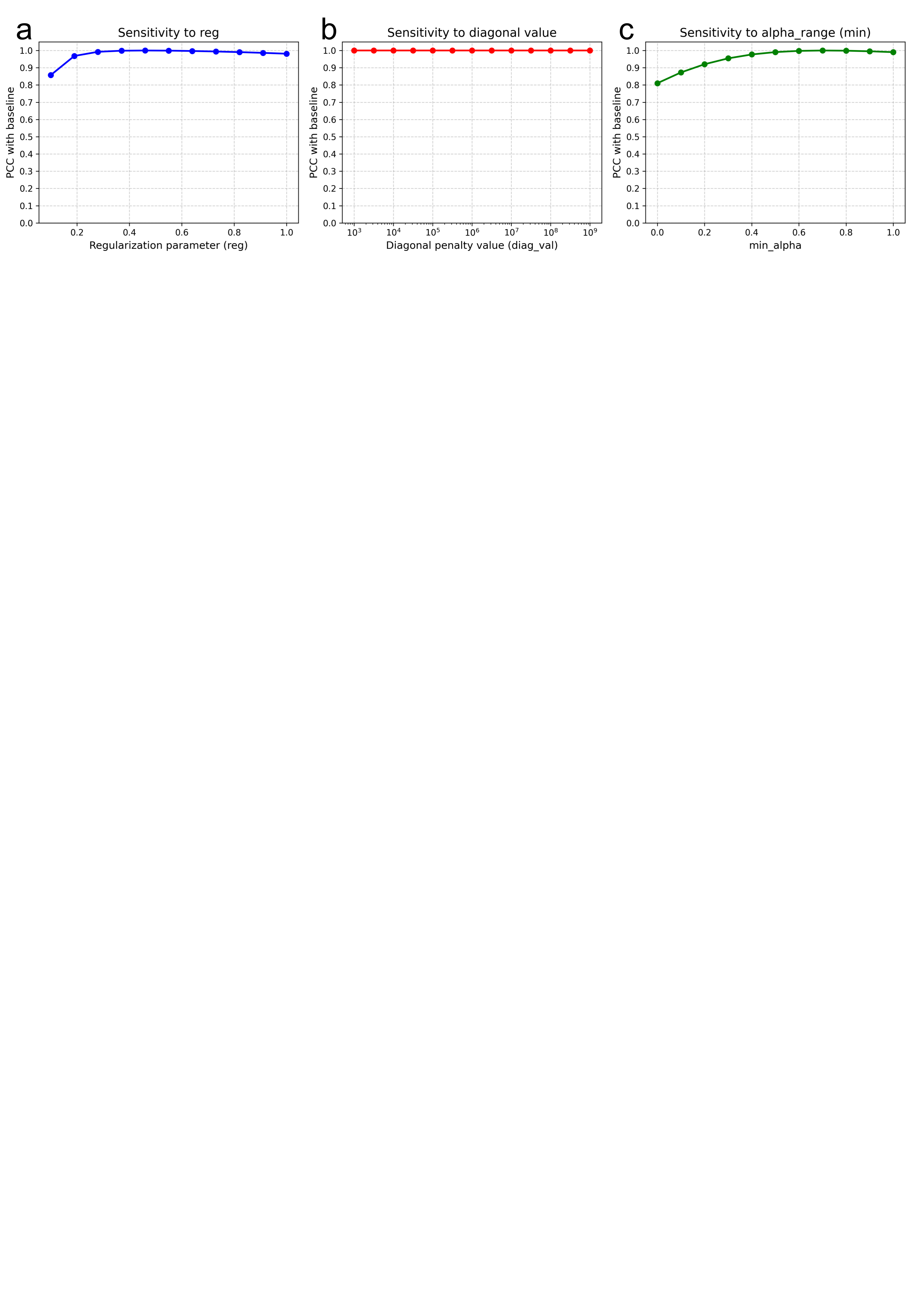


**S16 Fig. Sensitivity analysis of hyperparameters in the model.**

**a.** Sensitivity to the diagonal term parameter. The diagonal term parameter is varied from $10^{5}$ to $10^{9}$, with $10^{7}$ set as the baseline. For each value, the corresponding optimal transport (OT) matrix is computed, and the Pearson correlation coefficient (PCC) is calculated between this matrix and the OT matrix obtained under the baseline parameter.

**b.** Sensitivity to the regularization parameter reg. The regularization parameter reg is varied from 0.1 to 1 in steps of 0.1. For each reg value, the corresponding OT matrix is computed, and the PCC between this matrix and the OT matrix obtained with reg = 0.1 (used as the baseline) is calculated.

**c.** Sensitivity to the alpha_range (min) parameter. The alpha_range (min) parameter is varied from 0.1 to 1, with 0.7 set as the baseline. For each value, the corresponding OT matrix is computed, and the PCC is calculated between this matrix and the OT matrix obtained under the baseline parameter.
