# Supplementary material for: StPedf: Cell trajectory inference of spatial transcriptomics via spatial proximity embedding and spatial density-adaptive fusion
Source: PLoS Comput Biol. 2026 Jun 5;22(6):e1014346. doi: 10.1371/journal.pcbi.1014346 (PMC13240877; doi:10.1371/journal.pcbi.1014346)
Supplement: S17 Fig — a. Inferred spatial trajectories obtained after removing the adaptive explicit spatial fusion module on the Ambystoma mexicanum telencephalon regeneration dataset. b. Pseudo-spatiotemporal map obtained after removing the adaptive explicit spatial fusion module on the Ambystoma mexicanum telencephalon regeneration dataset. c. Inferred spatial trajectories obtained after removing the adaptive explicit spatial fusion module on the ICC tumor dataset. d. Pseudo-spatiotemporal map obtained after removing the adaptive explicit spatial fusion module on the ICC tumor dataset. (DOCX) [file pcbi.1014346.s025.docx]

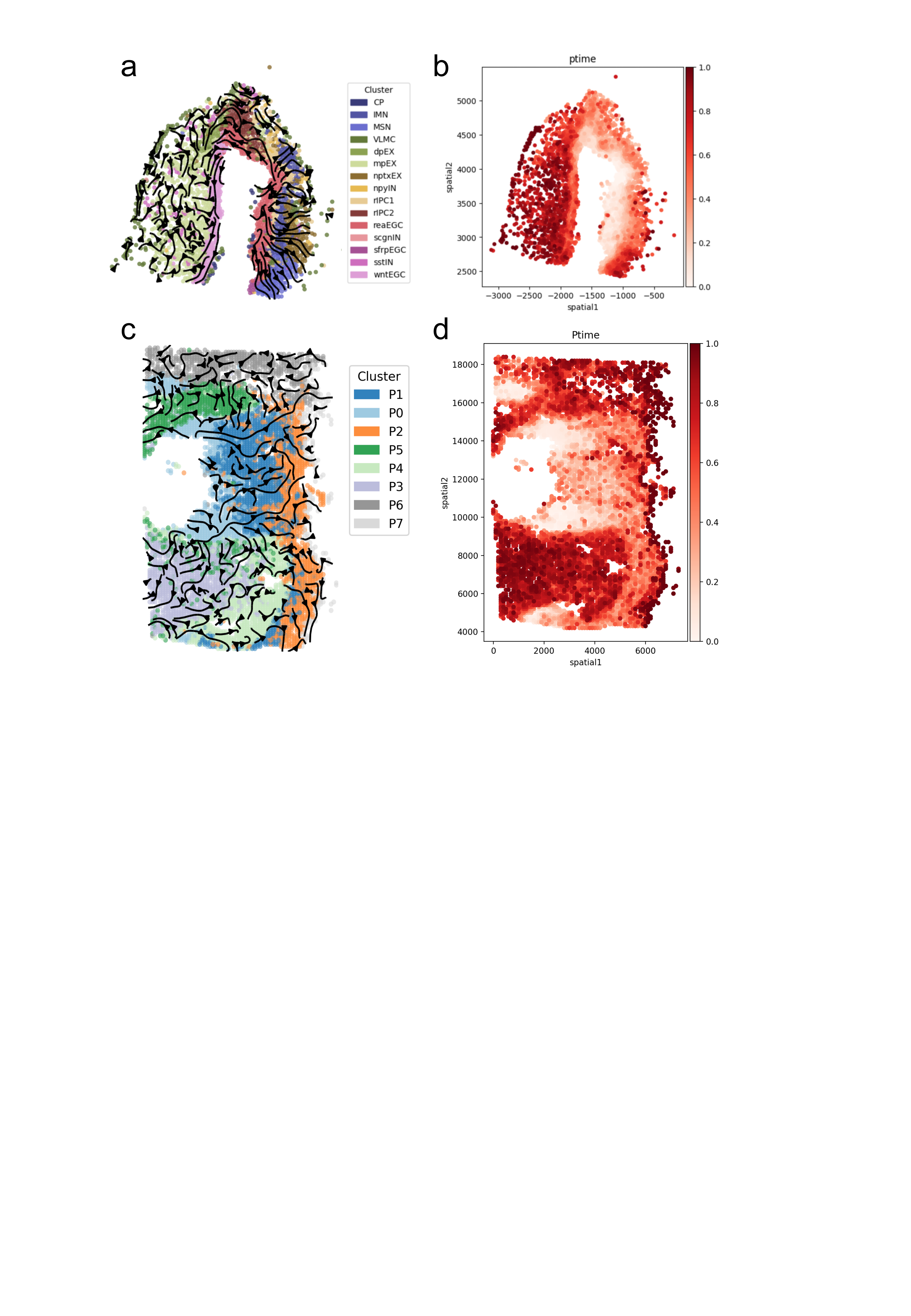


**S17 Fig. Ablation study of the adaptive explicit spatial fusion module in real datasets.**
**a.** Inferred spatial trajectories obtained after removing the adaptive explicit spatial fusion module on the *Ambystoma mexicanum* telencephalon regeneration dataset.
**b.** Pseudo-spatiotemporal map obtained after removing the adaptive explicit spatial fusion module on the *Ambystoma mexicanum* telencephalon regeneration dataset.
**c.** Inferred spatial trajectories obtained after removing the adaptive explicit spatial fusion module on the ICC tumor dataset.
**d.** Pseudo-spatiotemporal map obtained after removing the adaptive explicit spatial fusion module on the ICC tumor dataset.
