# Supplementary material for: StPedf: Cell trajectory inference of spatial transcriptomics via spatial proximity embedding and spatial density-adaptive fusion
Source: PLoS Comput Biol. 2026 Jun 5;22(6):e1014346. doi: 10.1371/journal.pcbi.1014346 (PMC13240877; doi:10.1371/journal.pcbi.1014346)
Supplement: S18 Fig — a. Comparison of Spearman correlation coefficients between the full model (StPedf) and the variant without clustering loss across four simulated datasets. b. Comparison of Kendall correlation coefficients between the full model (StPedf) and the variant without clustering loss across four simulated datasets. (DOCX) [file pcbi.1014346.s026.docx]

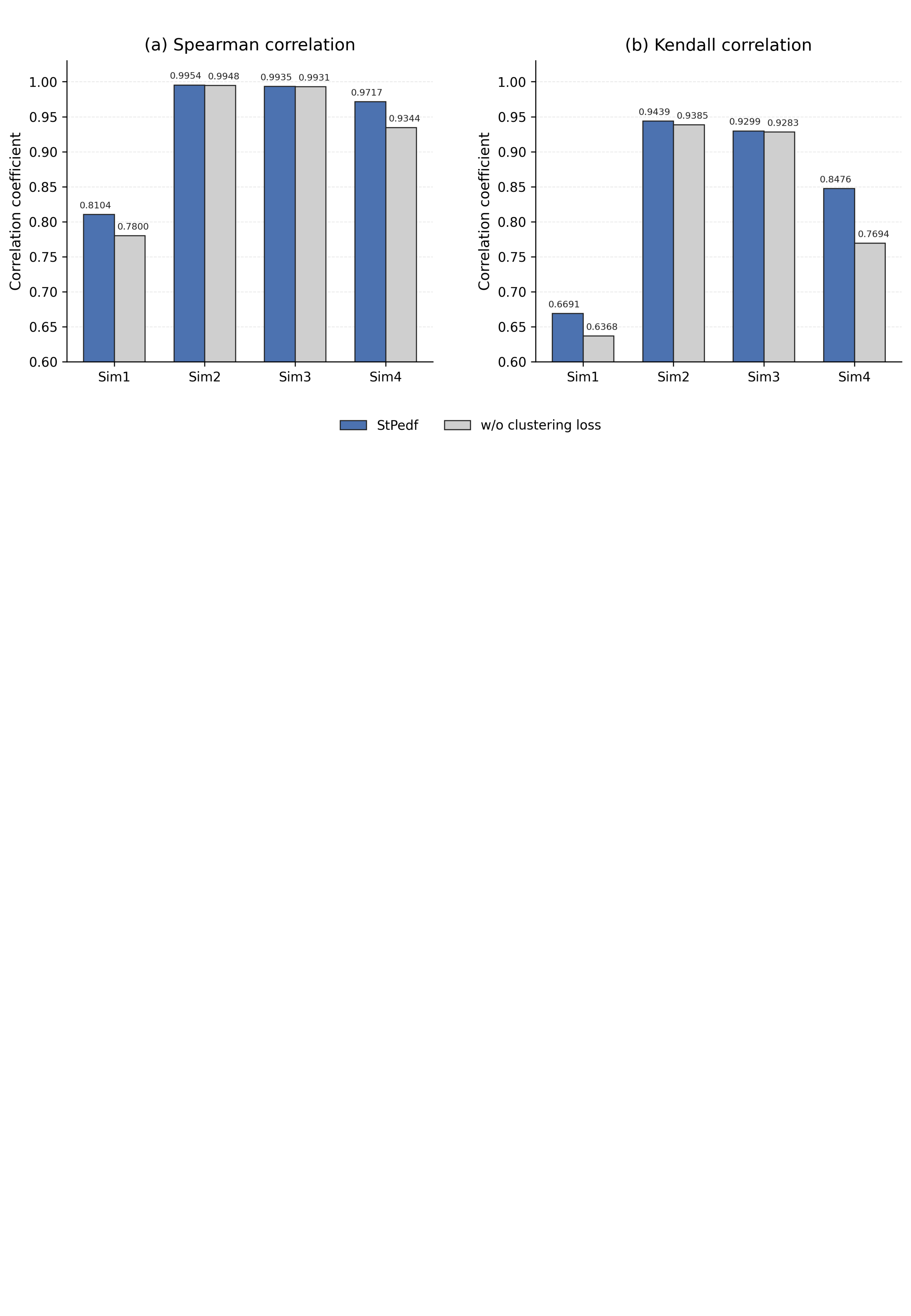


**S18 Fig. Ablation study of the clustering loss on simulated datasets.**

**a.** Comparison of Spearman correlation coefficients between the full model (StPedf) and the variant without clustering loss across four simulated datasets.

**b.** Comparison of Kendall correlation coefficients between the full model (StPedf) and the variant without clustering loss across four simulated datasets.
